# Quantification Challenges in Polymer Analysis in Urban Runoff and Wastewater using Pressurized Liquid Extraction and Double-Shot Pyrolysis-Gas Chromatography-Mass Spectrometry

**DOI:** 10.1021/acs.analchem.5c01170

**Published:** 2025-07-07

**Authors:** Daniele Martuscelli, Jonas B. Jensen, Luca Solari, Simona Francalanci, Peter Christensen, Jan H. Christensen

**Affiliations:** 1 Department of Civil and Environmental Engineering, 9300 University of Florence, Via di Santa Marta, 3, Florence 50139, Italy; 2 Analytical Chemistry Group, Department of Plant and Environmental Science, Faculty of Science, University of Copenhagen, Thorvaldsensvej 40, Frederiksberg 1871, Denmark

## Abstract

Microplastics are persistent environmental pollutants with potential risks to ecosystems and human health. This study optimized methods for isolating and quantifying polyethylene (PE), polyethylene terephthalate (PET), polypropylene (PP), and polystyrene (PS) in environmental water and wastewater samples using pressurized liquid extraction (PLE) combined with pyrolysis-gas chromatography/mass spectrometry (Py-GC/MS). The optimized two-step PLE protocol, consisting of methanol pre-extraction at 100 °C followed by tetrahydrofuran (THF) at 180 °C, achieved MP recoveries of 43–58%. Calibration curves were established using both solubilized and solid MP standards, with solubilized calibrations providing higher accuracy for PET and PP. Py-GC/MS conditions were optimized at 625 °C for 40 s to ensure maximum sensitivity and reproducibility. PE and PET were identified as the dominant MPs in the wastewater samples, with concentrations of 99.4 ± 71.8 μg/L and 16.2 ± 13.3 μg/L in Avedøre, and significantly higher levels of 749.0 ± 200.0 μg/L and 56.7 ± 22.6 μg/L in Pontedera. PP and PS were detected at lower concentrations, with PP ranging from 8.2 ± 4.2 μg/L to 16.9 ± 6.5 μg/L, and PS from 2.2 ± 1.5 μg/L to 8.4 ± 2.6 μg/L. Our results highlight the challenges of MP analysis in water samples, emphasizing the impact of key method parameters (e.g., MP isolation, pyrolysis, and calibration) on analytical reliability. The proposed method enhances MP detection in different water matrices, offering a reliable approach for routine environmental monitoring. Future efforts should focus on refining protocols to improve accuracy and precision and advancing standardization to ensure consistent and accurate MP quantification.

## Graphical abstract

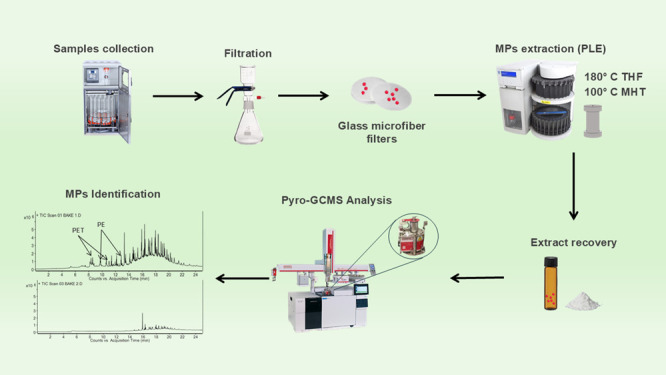

## Introduction

Microplastics (MPs), defined as plastic particles smaller than five millimeters, have become a significant environmental concern due to their widespread presence and persistence in ecosystems. When ingested by animals, MPs can cause physical harm, toxic exposure, and bioaccumulation, ultimately entering the human food chain.^1,2^ This has raised increasing concerns about their impact on human health, as studies continue to report their presence in food and water sources.^3,4^

Numerous studies have detected MPs across environments, from urban areas to remote regions like the North Atlantic, highlighting the global scale of this issue.^5,6^ While much research has focused on marine environments, the roles of rivers, runoff, wastewater, and overflows in MP pollution remain poorly understood.^7^ A key challenge is the lack of standardized analytical protocols, leading to inconsistent and difficult-to-compare results across studies. Despite the development of methods for isolating and quantifying MPs, a universal standard is still lacking, with evolving methodologies failing to establish consistent benchmarks.^8,9^

MP analysis involves two key steps: isolation from environmental matrices and quantification. Traditional isolation techniques, such as density separation (e.g., NaCl or high-density solutions), can be inefficient due to variations in MP density or adhesion to particulate matter, leading to biased quantification.^10–12^ Chemical digestion with oxidative agents (NaClO, H_2_O_2_), bases (NaOH, KOH), or acids (HCl, HNO_3_) is also commonly used to remove organic material, but it can degrade certain polymers and hinder the detection of plastic additives or adsorbed micropollutants.^13–15^

To improve isolation efficiency, emerging methods such as the Oil Extraction Protocol (OEP), which exploits the oleophilic nature of plastics, have been tested in combination with density separation.^16^ Electrostatic and magnetic separation techniques have also been explored to selectively isolate MPs based on their electrical or magnetic properties.^17–19^ These alternatives aim to enhance recovery and reduce biases in MP quantification

Pressurized Liquid Extraction (PLE) has recently shown promising results for isolating MPs.^10,11^ PLE uses solvents at high pressure and temperature, enhancing MP extraction by keeping solvents in a liquid state at elevated temperatures, thereby increasing solubility. This method is particularly effective for isolating MPs from complex matrices like sediments and biological tissues and can detect even low MP concentrations.^12^

MP quantification of solid or solubilized MP samples can be performed with laser scanning or chromatography-based methods. Laser scanning analyzes MP spectra to visualize characteristics like shape, number, polymer type, and color but can struggle with very small particles. In contrast, chromatography-based methods, especially Pyrolysis- gas chromatography/mass spectrometry (Py-GC/MS), are better for analyzing MPs of all sizes. Py-GC/MS breaks down polymers through pyrolysis and detects thermal degradation products and reliable quantification requires monitoring of selective products at specific mass-to-charge (*m*/*z*) ratios. This method also reduces the need for extensive sample preparation as it can be performed on both solid and liquid (solubilized) samples, but sample cleanup is beneficial to avoid quantification biases^1,13–16^

Py-GC/MS is used not only for identifying and quantifying MPs but also for detecting associated micropollutants, such as plastic additives or environmental pollutants absorbed by MPs.^17,18^ Common pyrolyzers include Curie Point, Micro Furnace, and filament types, which, despite varying heating methods, consistently produce reliable results for MP quantification, enabling cross-study comparisons.^19^ However, quantifying MPs in the low range of 25–385 μg poses challenges in preparing both MP standards and samples. Calibration curves can be created by diluting MPs with inert materials like glass microfibers or silica for even pyrolysis distribution,^19,20^ but this process is prone to errors during preparation.

Alternatively, MP solubilization can be performed prior to analysis using solvents like dichloromethane (DCM) or tetrahydrofuran (THF). It has been demonstrated that^11^ PLE with DCM effectively solubilizes polyethylene (PE) and polypropylene (PP), while another study^21^ used THF for polyethylene terephthalate (PET). For polymers such as PE and PP, where solubilization is difficult, MPs are analyzed in solid form.^22^ Selecting appropriate solvents to solubilize various MP mixtures is crucial. One strategy that will be tested in this study involves using Hansen solubility parameters to evaluate the solubilization potential of organic solvents on the different MP polymers treated in this study.^23^

The creation of solid or liquid calibration curves is essential for reliably quantifying MPs. Dierkes et al. (2019) reported limits of quantification (LOQs) between 1.4 and 1.6 μg for PE, PP, and polystyrene (PS), calculated from the standard deviation of blank samples. Matsueda et al. (2021) found higher LOQs for PET, around 10 μg, reflecting the varying challenges in quantifying different polymer types.

PLE was selected in this study for MP due to its suitability for handling complex matrices rich in organic matter, such as wastewater and combined sewer overflows (CSOs). Unlike conventional density separation and filtration, PLE allows for selective solubilization of MPs while simultaneously removing dissolved organic matter through controlled temperature and solvent polarity. It is high degree of automation reduces contamination risk and improves reproducibility between replicates. Moreover, the dry residues obtained from PLE with trapping material in the collection vials (in this study hydromatrix) are fully compatible with Py–GC/MS, enabling an efficient transition from extraction to quantification without additional filtration steps.

This study aims to refine MP quantification by optimizing PLE for particulate matter extraction and evaluating two calibration approaches for Py-GC/MS analysis of PE, PP, PS, and PET. It focuses on selecting optimal solvents, optimizing pyrolysis conditions, and assessing key PLE parameters (pressure, temperature, and extraction cycles) to enhance MP recovery from complex water matrices. Additionally, Py-GC/MS settings (pyrolysis temperature, split ratio, and flow conditions) will be fine-tuned to improve sensitivity and reproducibility. By refining these methodologies, the study seeks to establish more reliable and standardized protocols for MP isolation and quantification.

Although PLE has been applied for to solid samples, its application to filtered particles from aqueous samples remains limited. In this study, we adapted a dual-solvent PLE protocol, using methanol for matrix cleanup and THF for polymer solubilization, and collection of MPs on a solid material in the PLE collection vial. The solid material is then analyzed by a double-shot Py-GC/MS method for MP quantification. Calibration in both solid and liquid phases, enabled detection of PE, PP, PET, and PS in a realistic concentration range of 200 ng to 10 μg.

The method was applied to environmental samples collected from CSOs, a highly relevant but under-investigated source of MP pollution. These episodic discharges, triggered by intense rainfall, can introduce large volumes of particulate matter into receiving waters and are recognized as major contributors to urban MP loads. To date, no quantitative studies employing thermal analysis techniques have targeted these complex matrices. This study provides the first chemically resolved MP concentrations from CSO events, contributing novel insights into the role of urban drainage in plastic pollution.

## Material and Methods

### Reference Standards and Calibrations

MP reference standards used for calibration and recovery experiments included PE, PP, PET, and PS in pellet form, provided by CC Plast (Hillerød, Denmark), with declared purities >99%. Pellets were cryo-milled using a TissueLyser II (QIAGEN) after liquid nitrogen immersion and sieved to obtain particles >100 μm, in agreement with environmental survey thresholds.

Calibration was performed using both solubilized and solid-phase MP standards. For solubilized calibrations, MPs were dissolved in solvent mixtures optimized per polymer type (e.g., 20:80 octanol:toluene for PE/PP and 70:30 chloroform:TFA for PET/PS), heated (100–130 °C) to assist dissolution, and evaporated at 100 °C overnight. Aliquots of the resulting dry film, corresponding to 200 ng to 10 μg of polymer, were transferred into quartz pyrolysis vials (Gerstel) using preheated glass syringes.

For solid-phase calibration, the same mass range (200 ng–10 μg) of cryo-milled MPs was manually mixed with 2–3 mg of Hydromatrix and spiked with 0.95 μg of 4-fluorostyrene (Polymer Source), used as internal standard. The standard was solubilized in THF before addition. Mixing was performed manually using a metal spatula for 4–5 min to ensure homogeneity prior to loading into pyrolysis vials.

Limits of detection (DL) and quantification (LOQ) were determined from blank sample analyses and are reported in the Supporting Information (SI11)

### Pressurized Liquid Extraction

PLE was used to isolate MPs while minimizing interference. Samples were filtered (GB-140, 7 μm), dried, folded, and placed in 33 mL ASE-200 cells packed with Ottawa sand (1 cm from the top).

A two-step PLE method was optimized: a 100 °C methanol pre-extraction to remove micropollutants, followed by THF extraction at 180 °C. Key parameters: 1500 psi, 5 min static time, 3 methanol cycles, 2 THF cycles, flush volumes (MeOH 45%, THF 80%), 75 s purge. Extracts were collected in 80 mL vials with preheated Hydromatrix (450 °C).

PLE efficiency was assessed via hydrothermal extraction (180 °C, 16 h, 1 °C/min increase) and by weighing vials before/after solvent evaporation. Two methods were tested: (1) three rinses (50% volume, combined) and (2) one rinse (150% volume). A four-cycle extraction was also evaluated for full polymer recovery

### Py-GC/MS Method: Development and Final Protocol

Py-GC/MS method was developed and optimized for MP analysis, following an initial protocol where 5 mg of milled MP sample were placed in pyrovials and analyzed in a Gerstel TDU 2 (thermal desorption unit). The method employed a double-shot approach: thermal desorption at 300 °C to remove volatile and semivolatile compounds, followed by pyrolysis at 625 °C for MP degradation and analysis of pyrolyzates.

The initial method^10^ applied pyrolysis at 600 °C for 20 s, injecting pyrolysis products at a 1:20 split ratio, followed by another 1:20 split (total 1:400 split). A split vent flow controlled the transfer through the TDU, while a second split was applied during column injection. The temperature between the pyrolysis unit and the injection port was maintained at 300 °C. Mass spectra were acquired on an Agilent 5975C inert XL MSD, with an interface temperature of 320 °C.

To improve sensitivity and prevent MSD saturation, particularly for PS, the split ratio was refined. The final optimized method maintained the double-shot approach, with thermal desorption at 300 °C (100 s hold) followed by pyrolysis at 625 °C (40 s hold, plus 1 min postpyrolysis transfer). Helium was supplied at 51.1 mL/min, with a 1:10 split ratio and a 10 mL/min purge flow. The CIS (cooled injection system) trapped analytes at 30 °C, then ramped to 300 °C at 12 °C/s, holding for 3 min before column transfer.

Separation was performed on an Agilent 7890A GC equipped with a Zebron ZB5 column (5% phenyl methyl siloxane, 30 m × 250 μm × 0.25 μm). The oven program started at 35 °C, ramping at 15 °C/min to 320 °C, followed by a 6 min hold. The helium flow was 1.1 mL/min, with detection in SIM mode (six ions per retention time window, 30 ms dwell time, 4.89 scans/s) or scan mode (*m*/*z* 10–400, 3.66 scans/min). The ion source and quadrupole were kept at 230 and 150 °C, respectively.

Through this methodological refinement, the final Py-GC/MS protocol ensured detection of all polymer types, optimizing split ratios to prevent signal saturation while maintaining high analytical sensitivity and reproducibility.

The method was validated using six-point calibration curves (200 ng–10 μg) with replicates (n = 2–3), internal standard correction, blank vials between samples, and preanalysis cleaning of pyrolysis interfaces. R^2^ values, 95% CI, and carryover tests are provided in SI6–SI8.

### Peak Identification, Detection, and Quantification

For MP polymer identification, specific indicator compounds are needed for each plastic type. In py-GC/MS analysis, PS is typically identified and quantified by styrene, α-methylstyrene, and cumene, while PET is quantified by dimethyl terephthalate and bis­(2-hydroxyethyl) terephthalate. PE is recognized by linear alkenes and alkanes such as 1-octadecene and 1,14-tetracosadiene, and PP by 2,4-dimethyl-1-heptene and 2,4-dimethyl-1-decene.

Reference standards were analyzed, and pyrograms were compared with literature data for accurate identification. Styrene (*m/z 104*) was used for quantifying PS, vinyl benzoate (*m/z 105*) for PET, 1-decene (*m/z 83*) for PE, and 2,4-dimethyl-1-heptene (*m/z 126*) for PP.

Table S2 lists the plastic polymers, target pyrolyzates, and corresponding *m*/*z* values used as quantified and qualified ions. SIM data files from the py-GC/MS were processed in Masshunter’s MS Quantitative Analysis software, with manual adjustments to ensure consistency.

## Result and Discussion

### Homogeneity of MP

Sample homogeneity is a key challenge in MP analysis.^12^ For particles >100 μm, high sample weights are typically required. Thermo-analytical methods use 10–50 mg, but our study was limited to 5–10 mg due to pyro vial capacity. Aggregate formation postextraction further reduced homogeneity, with polymers sometimes repolymerizing upon solvent evaporation, forming fragile aggregates atop the Hydromatrix layer, which were disaggregated using a hand-held miller.

To enhance homogeneity and reduce statistical uncertainty, we compared samples with and without homogenization (Figure S4). Aliquots (5 mg) were analyzed, the maximum workable in 10 mg pyrolysis cups. Initially, direct PY-GC/MS analysis was used. A mixing step with stainless steel spheres in a Reax mixer (overnight) was then tested. The most effective method was manual mixing (4–5 min with a spoon), previously reported but not quantified for effectiveness.

Figure S4 shows that manual mixing significantly improved reproducibility, reducing relative standard deviation (RSD) from 0.95% to 0.14% (2 μg/mg sample) and from 0.94% to 0.22% (160 μg/mg sample). Proper grinding/homogenization is essential for reliable MP analysis, ensuring uniform particle distribution and lower RSD in technical replicates.

### Polymer Solubility and Handling Challenges

The solubility of MPs varied significantly among polymers, with PS dissolving easily while PE and PP required heating and specific solvent combinations. Further details on polymer solubility and handling challenges are provided in the Supporting Information (SI8).

### PLE for Isolation of MPs from Sample Matrix

The two-step PLE method began with a pre-extraction using methanol at 100 °C to remove organic contaminants that could otherwise interfere with subsequent MP analysis. This step was followed by an extraction with THF at 180 °C to solubilize MPs effectively. Figure 1 presents the peak areas for PS and PE obtained during sequential extractions, highlighting the method’s selectivity.

**Figure 1:**
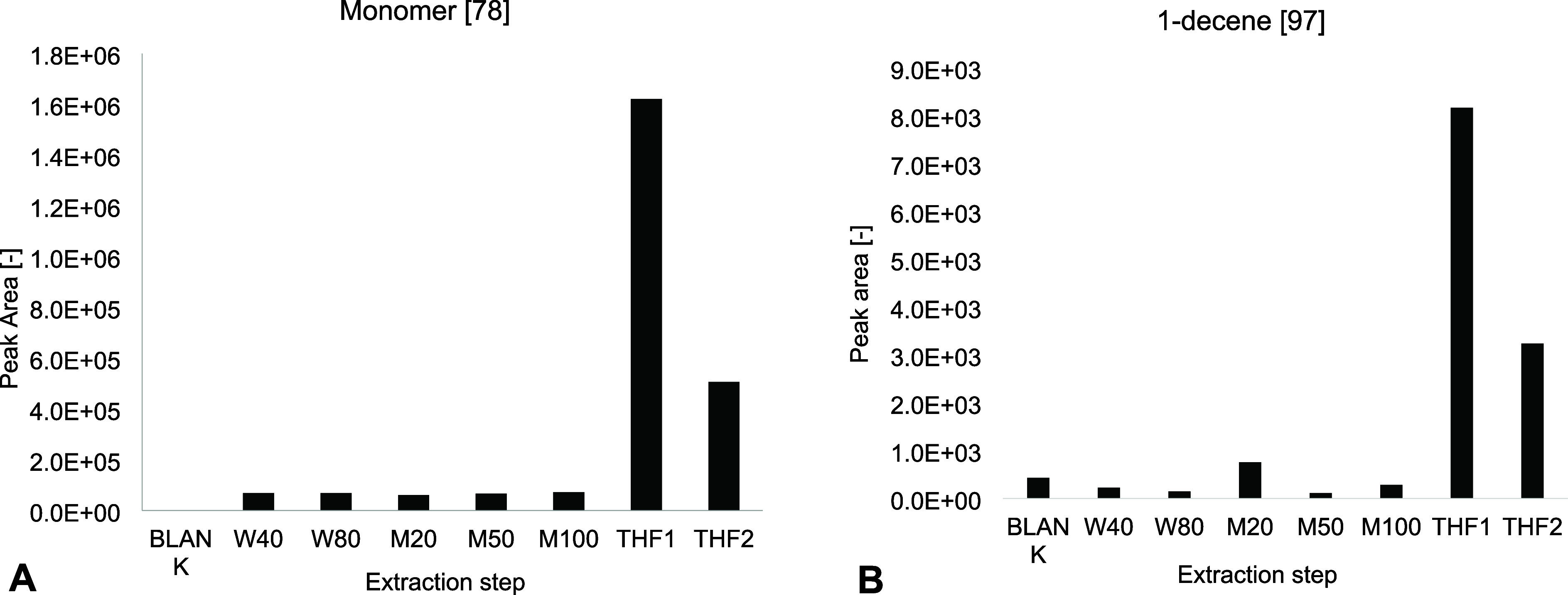
Peak area of representative compounds for PS (styrene, *m*/*z* 78) (A) and PE (i-decene, *m*/*z* 97) (B) after sequential extraction conditions. The blank represents the extraction of a PLE cell without spiking with MPs using THF at 180 °C. W40 and W80 refer to Milli-Q water extractions at 40 and 80 °C, respectively. M20, M50, and M100 represent extractions with 20, 50, and 100% MeOH in Milli-Q water at 100 °C. THF1 and THF2 denote two consecutive THF extractions at 180 °C.

Results demonstrated that water and methanol extractions, regardless of temperature, did not solubilize the polymers, as peak areas remained like blank values, indicating minimal polymer extraction. In contrast, the first THF extraction yielded significantly higher MP concentrations, confirming effective polymer isolation with little need for a second THF extraction where the peak area was around 30% compared to the first extraction peak area for both polymers, PS and PE. This outcome confirms that the methanol pre-extraction step can successfully remove potential organic interferences without compromising MP recovery. However, at the same time it requires full solubilization of the plastic polymers with the extraction solvent (in this case THF) at the extraction temperature 180 °C, and this has not been confirmed with PET or PP.

Each extracted fraction was collected and analyzed by Py-GC/MS to quantify MPs. Recovery was determined by comparing the peak areas of PS, PE, PP and PET in the Hydromatrix collected from PLE with those in the extract obtained by 16 hours of heating in a metal cell, yielding recoveries between 43% and 58% for the different MPs (43% for PS, 46% for PET, 44% for PE, and 58% for PP).

A second evaluation method involved weighing the Hydromatrix in the collection cells before and after PLE. Results for PS confirmed that no extraction occurred during the MeOH pre-extraction, while the THF extract recovered 87 ± 1% (n = 2) of the PS initially added to the PLE cell.

Although previous studies have used multistep solvent extraction with PLE, the present method introduces key improvements that improve both versatility and environmental relevance. The dual-phase calibration strategy - employing both solubilized polymer films and solid-phase mixtures with Hydromatrix - offers performance across a range of sample types, improving method transferability. More importantly, the method was successfully applied to samples from CSO events, which constitute episodic but intense releases of MPs into the aquatic environment.

### Influence of Temperature and Time on Pyrolysis Efficiency

Before analyzing real samples, we focused on selecting the pyrolysis temperature to ensure complete polymer degradation and consistent signals responses across samples with identical polymer quantities. Various pyrolysis technologies exists,^24–26^ and previous studies^11^ have identified effective pyrolysis conditions to minimize interlaboratory discrepancies.

A preliminary study on PS pyrolysis duration found that extending the time from 20 to 40 s resulted in higher peak areas. Based on this, a pyrolysis time of 40 s was selected for further temperature optimization without additional refinements.

The effect of pyrolysis temperature on the abundance of target pyrolyzates was evaluated for all four MPs. Six temperatures, ranging from 550 to 675 °C, were tested using a 40-s pyrolysis time, a 1:50 split ratio, and a CIS transfer temperature of 250 °C. Each temperature was tested in triplicate across three batches, with batch orders varied (low to high, high to low, and random), and cleaning steps performed between runs.

Results (Figure 2) showed that PE pyrolysis was most efficient at 625 °C, with C10–C14 α-alkene peaks nearly doubling compared to other temperatures. A similar trend was observed in another study,^11^ where selected 600 °C to maximize PE detection in biosolid matrices using Pyr-GC/MS. PET pyrolyzates showed high temperature sensitivity; while vinyl benzoate remained constant, biphenyl and benzophenone increased 8–10 times between 550 and 675 °C, in line with thermal decomposition profiles described in pyrolysis reviews.^1,9,27^ PP pyrolyzates, particularly the early eluting trimer “2,4-dimethyl-1-hept-1-ene(126)” and the “2,4,6,8-tetramethyl-1-undecene (111)” peaks, remained stable between 550 and 625 °C but decreased at higher temperatures. For PS, styrene levels remained consistent across temperatures, while the PS-dimer “3-buten-1,3-diyldibenzene (91)” and 2,5-diphenyl-1,5-hexadiene (234) showed slight decreases at higher temperatures.

**Figure 2:**
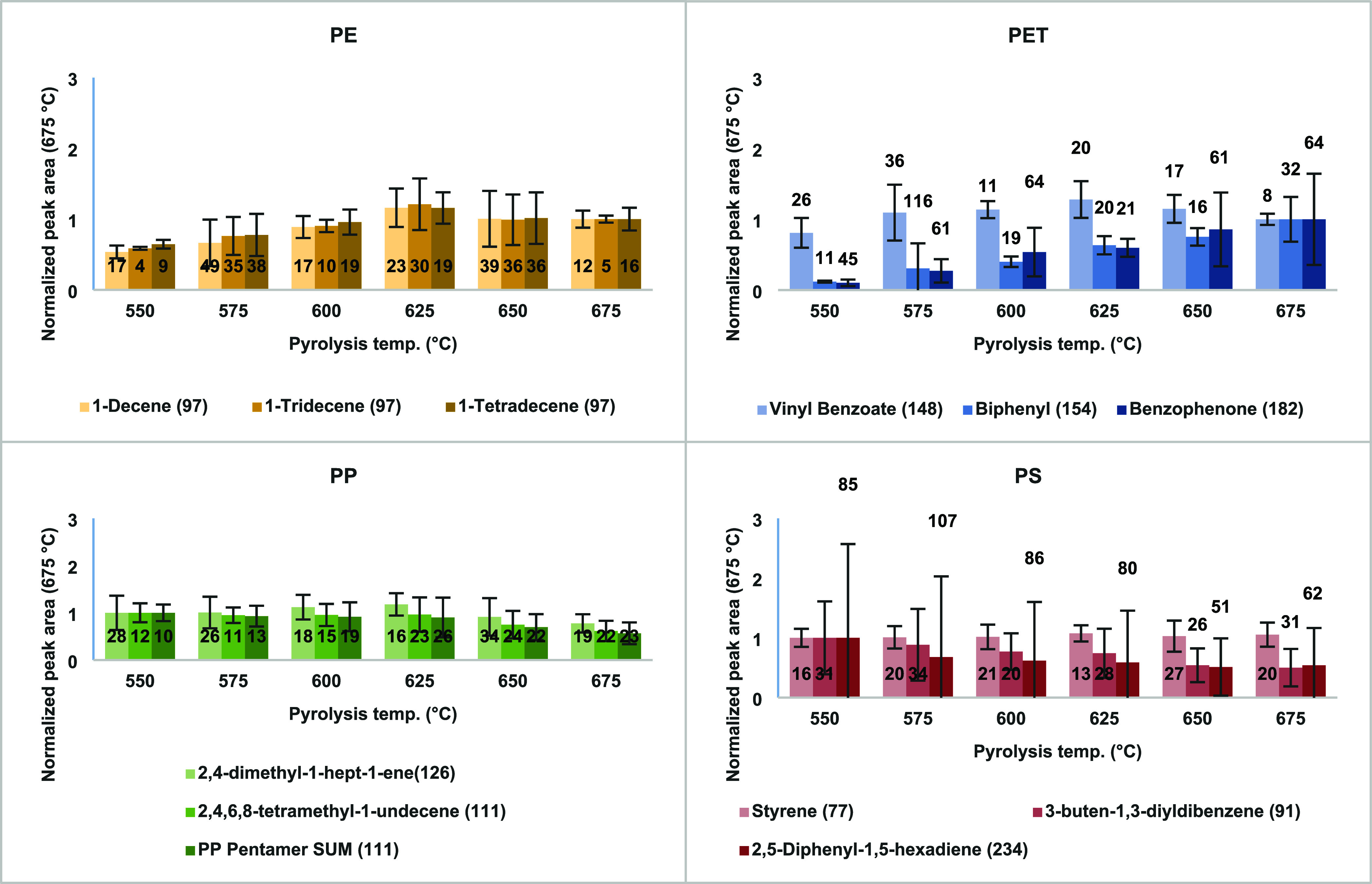
Normalized peak areas of selected pyrolyzates for PE, PET, PP, and PS at different pyrolysis temperatures. The *x*-axis represents the pyrolysis temperature (°C), while the *y*-axis shows the normalized peak areas relative to 675 °C. Pyrolyzate names and their corresponding extracted ion (*m*/*z*) values are indicated in the legends below each chart. Error bars represent the 95% confidence interval (*n* = 3), with relative span percentages displayed above or within the bars for each temperature condition.

Systematic comparison of six pyrolysis temperatures between 550 and 675 °C showed compound-specific responses, such as a sharp increase in biphenyl and benzophenone from PET, and a decline in PP trimers above 625 °C. The selected 40 s pyrolysis time was validated through preliminary tests on PS and is consistent with results from another study^11^ who reported no signal suppression using 20–40 s in their double-shot setup.

### Influence of Inorganic Matrix in Pyrolysis-Vials

Under standard pyrolysis conditions without a matrix, PS pyrolysis primarily produces styrene (monomer), 3-butene-1,3-diyldibenzene (dimer), and 5-hexene-1,3,5-triyltribenzene (trimer), with the highest signals observed for the monomer, trimer, and dimer. Additional byproducts include α-methylstyrene, benzene, and xylene.^28,29^

The presence of an inorganic matrix significantly alters the yield of these characteristic PS pyrolysis products, even with identical PS amounts. For PET, the addition of silica gel caused a near-complete suppression of diagnostic peaks, suggesting that silica gel interacts with analytes or decomposes key markers such as benzoates and terephthalates.^30^

Comparative tests using three inert matrices and PS showed varying effects on styrene peak areas. Silica gel caused the most deviation, glass beads produced values closer to pure PS, and Hydromatrix provided the most stable pyrolysatogram (Figure S6), making it the preferred inorganic matrix in our study. These results is comparable with those obtained in a similar study,^30^ who observed a reduced signal intensity of PS marker compounds when pyrolyzed with silica. However, our study extends this comparison by simultaneously evaluating three matrices under controlled conditions and by monitoring multiple PS markers (monomer, dimer, trimer). Notably, Hydromatrix provided the best reproducibility, measured as relative standard deviation (RSD), with values as low as 0.7% for styrene, compared to 31.9% with silica gel, 9.2% with glass beads, and 7.5% with no matrix. Similarly, lower RSDs were observed for the PS dimer (4.9%) and trimer (14.0%) with Hydromatrix, confirming its superior consistency. These results contrast with the greater variability reported in a previous study^31^ for both alumina and silica (see Figure 3).

**Figure 3:**
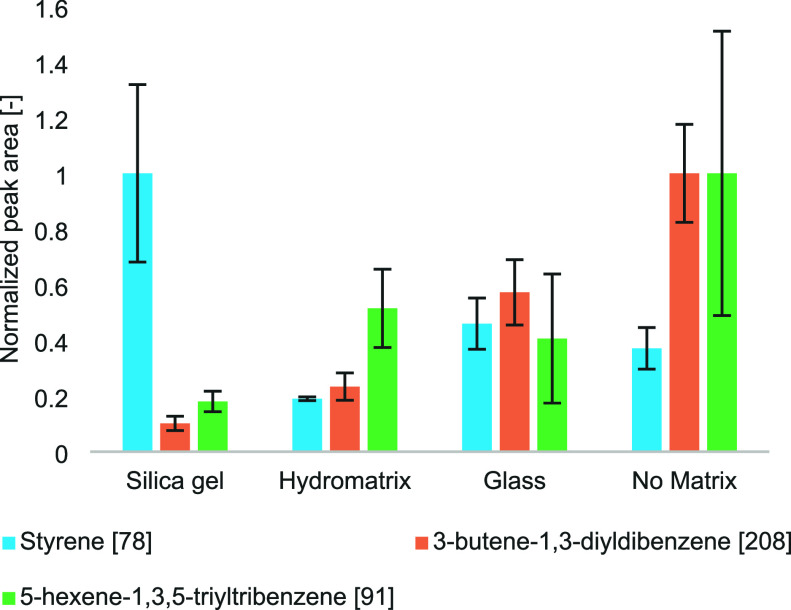
Influence of inorganic matrices on PS pyrolysis products. All values are normalized to the highest detected signal. Peak areas of styrene monomer (*m*/*z* 78), 3-butene-1,3-diyldibenzene (*m*/*z* 208) and 5-hexene-1,3,5-triyltribenzene (*m*/*z* 91) for PS pyrolysis with different inorganic matrices: silica gel, Hydromatrix, glass beads, and no matrix. Equal sample amounts were analyzed, with error bars representing relative standard deviations and mean peak areas shown. Silica gel yielded the highest peak area for monomer and dimer, while Hydromatrix ensured consistent results.

### Effects of Split Ratios and Flows and Temperatures in the TDU

A test was conducted to evaluate the impact of split ratios in the py-GC/MS method on selected pyrolyzate peak areas. Split ratios of 1:50, 1:25, 1:10, and splitless were tested with single replicates, and the MS operated in scan mode. Results (Table S3) show that for PE, peak areas did not scale proportionally with decreasing split ratios, remaining under a 2-fold increase for most pyrolyzates, except 1-tetradecene in splitless mode. PET pyrolyzates followed a similar pattern from 1:50 to 1:10, but in splitless mode, peak areas of vinyl benzoate and biphenyl increased 6–7 times, while divinyl terephthalate and benzophenone rose 25-fold. PP pyrolyzates, including the trimer and pentamer peaks, showed minimal change across split ratios. The PS styrene monomer remained stable, while the PS dimer, 2,5-diphenyl-1,5-hexadiene, and trimer increased similarly to PET’s divinyl terephthalate and benzophenone.

These results are consistent with other studies^19,32^ who observed that Py-GC/MS peak areas often increase with lower split ratios, though the response is not always linear due to saturation effects or secondary reactions among pyrolyzates. Moreover has been noted^32^ unexpectedly high responses of certain PET and PC markers under high load or mixed-polymer conditions, an effect also evident in our data, particularly for PET in splitless mode. Similarly, has been demonstrated^33^ stable responses for PP trimer and PS monomers under varied preparation conditions, supporting the stability observed in our study for these markers

Comparable conclusions were reported in another study,^34^ showing that high calibration cruve linearity (R^2^ > 0.995) in MP quantification required split ratio adjustment, specifically 100:1 for high-load and 10:1 for low-load samples, as a single calibration curve failed to maintain linearity for all polymers at a large mass range.

Compared to these studies, our results provide a more detailed polymer-specific analysis under varying split conditions, particularly highlighting the disproportionate signal increase of selected PET and PS pyrolyzates in splitless mode. This suggests that splitless injection can lead to overestimation of specific markers, especially in complex matrices or at high analyte loads, and should therefore be carefully validated for quantitative applications.

### Reduction of Carry-over

Following sample pyrolysis, the TDU hold time can be adjusted to stay warm for a set duration. This study tested 1 min (n = 2) and 5 min (n = 2) hold time to assess their impact on carryover. Pyro-vials containing a mixture of four MPs were analyzed using the double-shot method, followed by blanks to evaluate carryover. A 1:50 split ratio was applied with the MS in scan mode.

TDU hold time influenced both signal intensity and carryover for certain analytes. A longer hold time caused earlier elution of smaller analytes compared to the 1 min hold. For example, the PP trimer, the earliest eluting analyte, shifted by 1.26 min with a 5 min hold. Analytes eluting at 9 min or later were unaffected. Additional details are in Table S4 (left side).

Carryover signals of PE pyrolyzates in blanks showed minimal differences between hold times (Table S4, right side), with both resulting in 1–3% carryover, slightly lower with the 5 min hold. This trend was consistent for PP pyrolyzates and other early eluting analytes. However, later-eluting analytes—PS dimer, trimer, and 2,5-diphenyl-1,5-hexadiene— showed reduced carryover with the longer hold time.

The calibration curve for solubilized MP polymers was analyzed in three batches, progressing from lowest to highest concentration, with blank pyro-vials between samples to monitor and minimize carryover. Figure 4 shows carryover signals for selected pyrolyzates, used in calibration curves described in the next section. For PE, 1-decene showed the lowest carryover, while 1-dodecene and 1-tetradecene had higher levels. Carryover remained stable from 200 ng to 2 μg, but blanks following the 6 and 10 μg showed increased peak areas across all three pyrolyzates.

**Figure 4:**
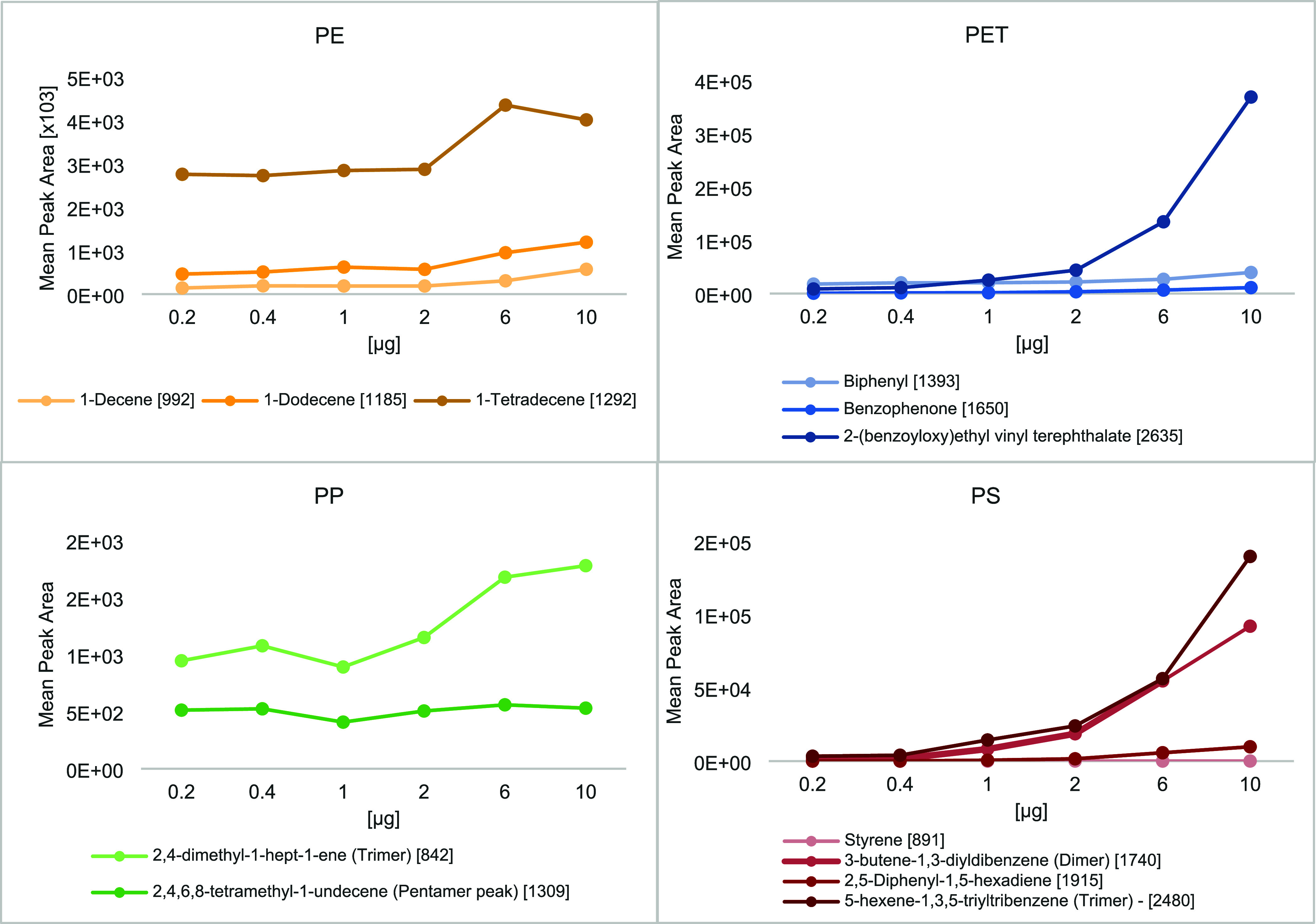
Mean peak area (*n* = 3) of blank samples analyzed between consecutive calibration curve points, with samples run in ascending concentration order.

These observations are consistent with general recommendations from instrument manufacturers such as Gerstel, which advice extended hold times at high temperature in the TDU to reduce memory effects between injections, particularly for high-boiling analytes. However, such guidelines are qualitative and lack numerical quantification. In contrast, this study provides a systematic, quantitative evaluation of carryover reduction as a function of hold time for a range of MP pyrolyzates, offering a novel and practical contribution to method optimization.

The PP trimer followed this trend, while the first PP pentamer peak showed minimal carryover change. The biphenyl peak from PET had the highest initial value, doubling from 17,700 to 39,500 between the 200 ng and 10 μg samples, with benzophenone showing a similar pattern at lower levels. The larger PET pyrolyzate, 2-(benzoyloxy)­ethyl vinyl terephthalate, showed an 80-fold carryover increase from the lowest to highest concentrations. PS dimer and trimer followed a similar trend, while 2,4-diphenyl-1,5-hexadiene showed distinct peaks in blanks starting at 1 μg injections. The styrene monomer produced minimal carryover, appearing as integrated noise at 3.7 min.

In addition to carry-over effects, the influence of sample loading volume on response signal was tested, showing that smaller incremental loads resulted in a higher detector signal due to concentrated deposition at the vial bottom. Further details are provided in the Supporting Information (SI9).

### Comparison of Solid and Liquid Calibration Curve Methods

Calibration curves for the four MP polymers (PE, PET, PP, and PS) were prepared from both solubilized polymers and solid MP particles diluted in Hydromatrix (Table 1 and SI6–SI8). PE calibration curves showed minimal differences between the two forms. For PET, the solid calibration curve had steeper slopes for biphenyl and benzophenone, with a similar but less pronounced trend observed for PP. In contrast, the solubilized PS curve had a steeper slope than its curve based on solid MP particles diluted in Hydromatrix. Regression parameters for calibration curves, using fluoro-styrene as an internal standard, are presented in Table 1.

**Table 1:** Comparison of Second-Order Regression Parameters for Calibration Curves of Solubilized and Solid MP Polymers^a^

	**solution**							**solids**						
**polymer**	**a1**	**a2**	**R2**	**LOQ (μg)**	**ratio**		**CI(95)**	**a1**	**a2**	**R2**	**LOQ (μg)**	**ratio**		**CI(95)**
PE	12760	28.84	0.94	0.13	5.14	±	0.37	17530	–251	0.97	0.09	5.29	±	0.56
PET	27350	–847	0.95	0.45	0.19	±	0.03	119500	–6968	0.54	0.10	1.08	±	0.97
PP	47290	276	0.95	0.13	2.67	±	0.06	65040	93	0.97	0.09	2.82	±	0.09
PS	3711000	–187000	0.95	0.00	230.6	±	55.23	2650000	–98670	0.89	0.00	291.9	±	129.7

a The left side contains parameters for solubilized MP polymers, while the right side represents solid MP particles diluted in Hydromatrix.

Table 1 compares the regression parameters of solubilized (Solution) and solid-phase (Solids) MP calibration for each polymer, highlighting differences due to sample preparation and MP distribution.

For PE, both methods produced comparable results, with the solid calibration yielding a slightly higher a_1_ value (17530 compared to 12760 in solubilized calibration) and a marginally improved model fit (R^2^ = 0.97 vs 0.94). The ratio values were consistent, indicating that both approaches provide reliable quantification.

For PET, significant discrepancies were observed. The solubilized method exhibited a much lower a_1_ value (27350, whereas the solid calibration reached 119500) and a better model fit (R^2^ = 0.95 compared to 0.54), suggesting that the solid calibration introduces higher variability, potentially due to matrix effects or polymer degradation. Additionally, the solubilized approach showed lower and more stable ratio values (0.19 ± 0.03, in contrast to 1.08 ± 0.97 in the solid method).

For PP, moderate differences were found, with the solid calibration yielding a higher a_1_ value (65040 relative to 47290 in solubilized calibration). However, both methods produced similar determination coefficients (R^2^ = 0.97 and 0.95, respectively), and ratio values remained close (2.82 ± 0.09 for solid calibration and 2.67 ± 0.06 for solubilized calibration), indicating consistent quantification across approaches.

For PS, the solubilized calibration resulted in a higher a_1_ value (3711000, while the solid calibration reached 2650000) and a better model fit (R^2^ = 0.95 compared to 0.89). However, higher response variability in the solid method (291.9 ± 129.7 vs 230.6 ± 55.23 for solubilized calibration) suggests potential differences in pyrolysis behavior between calibration approaches.

Overall, while solid calibration was effective for PE and PP, solubilized calibration provided superior consistency for PET and PS, with higher determination coefficients and more stable ratio values. Given these findings, solubilized calibration was preferred for MP quantification in environmental samples.

### Validation Tests On Environmental Samples: Utterlev Mose Samples

The quantification of MPs in filtered water samples from Utterslev Mose (SI1 and Figure S2), using calibration curves from solubilized PE, PET, PP, and PS, highlight the variability in concentrations in polymer concentrations (Table 2). PS concentrations were near the detection limits (DL). PP concentrations were below LOQ in all samples. Notably, PP levels in tap water were comparable to those in environmental samples A, B, and C, which ranged from 0.7 to 1.2 μg/L.

**Table 2:** MP Concentrations in Water Samples From Utterslev Mose and Blanks^a^

	sample A [μg/L]			sample B [μg/L]			sample B [μg/L]			Milli-Q [μg/L]			tap water [μg/L]		
polymer	mean	±	CI(95)	mean	±	CI(95)	mean	±	CI(95)	mean	±	CI(95)	mean	±	CI(95)
PE	72.9	±	33.8	148.9	±	62.0	232.0	±	118.0	5.3	±	2.1	4.4	±	3.2
PET	63.7	±	0.5	99.6	±	19.4	135.1	±	66.9	0.4	±	0.5	0.2	±	0.1
PP	1.0	±	0.7	0.7	±	0.4	1.2	±	1.4	1.2	±	1.0	0.5	±	0.2
PS	1.2	±	0.7	3.1	±	1.3	5.1	±	2.3	0.0	±	0.0	0.0	±	0.0

a Concentrations of PE, PET, PP, and PS in water samples A, B, and C (*n* = 4) from Utterslev Mose, as well as Milli-Q and tap water blanks (*n* = 6), were quantified using calibration curves from solubilized MPs without an internal standard. Mean concentration and absolute 95% confidence interval are presented in μg/L. Superscripts indicate (a) below DL, (b) below LOQ, and (c) above the highest calibration curve point

These findings are consistent with recent studies reporting low detectability of PP and PS in environmental water samples. Research suggests that microsized PS particles are prone to degradation and dispersion in natural waters, which can affect their quantification reliability.^12^ Moreover, the low PP levels align with its limited solubility and high buoyancy, which affect its environmental presence and detectability.^35^

PET and PE concentrations were considerably higher compared to other MPs, with PET levels of 63.7, 99.6, and 135.1 μg/L in samples A, B, and C, respectively. The elevated PET concentrations correspond to findings in other studies, attributing its accumulation in aquatic environments to its high density and environmental persistence.^36^ Benzophenone has been shown to be a reliable marker for PET detection with consistent quantification results even in complex environmental matrices.^30^

PE showed the highest concentration among the tested MPs, with values of 72.9 ± 33.8 μg/L (sample A), 148.9 ± 62.0 μg/L (sample B), and 232.0 ± 118.0 μg/L (sample C). These findings reflect PE’s extensive use in consumer products and its strong environmental persistence.^37^ Its lower density compared to PET contributes to its widespread distribution in surface waters, where it persists despite natural degradation processes.^38^

Overall, these results demonstrate the diverse environmental behaviors of MPs depending on polymer type, with PET and PE showing higher concentrations in filtered water samples, consistent with literature. The use of specific quantifier ions such as benzophenone for PET, enhances detection accuracy and underlines the importance of rigorous analytical methods to accurately capture concentration variability across polymer types.

### Wastewater Samples

MP analysis in wastewater samples from Avedøre (Denmark) and Pontedera (Italy) (SI1 and Figure S1) revealed significant MP concentration differences, reflecting site-specific variations commonly reported in wastewater studies (Table 3). The quantification of MPs in samples from both Avedøre and Pontedera, was made using calibration curves from solubilized PE, PET, PP, and PS, considered more reliabeble compared to the solid calibration curve (Table 1). In Avedøre, the mean PE concentration was 99.4 μg/L, while Pontedera exhibited substantially higher levels at 749.0 μg/L. These elevated concentrations in Pontedera are likely to result from higher wastewater volumes and industrial discharges, which are known to contribute to increased MP loads in WWTP influents.

**Table 3:** MP Concentrations in WWTP Samples from Avedøre and Pontedera^a^

	Avedøre [μg/L]			Pontedera [μg/L]		
polymer	mean	±	CI(95)	mean	±	CI(95)
PE	99.4	±	71.8	749.0	±	200.0
PET	16.2	±	13.3	56.7	±	22.6
PP	8.2	±	4.2	16.9	±	6.5
PS	2.2	±	1.5	8.4	±	2.6

a PE, PET, PP, and PS concentration in wastewater samples (*n* = 5) from Avedøre and Pontedera were quantified using calibration curves based on solid MP particles. Values represent mean concentrations and 95% confidence interval (μg/L). Superscript: (a) below DL, (b) below LOQ.

PET concentrations varied between sites, with Avedøre at 16.2 ± 13.3 μg/L and Pontedera at 56.7 ± 22.6 μg/L. The higher PET levels in Pontedera may be attributed to its persistence and accumulation in wastewater systems.

PP concentrations were 8.2 ± 4.2 μg/L in Avedøre and 16.9 ± 6.5 μg/L in Pontedera. The buoyancy of PP likely contributes to its retention in wastewater, resulting in moderate but consistent effluent levels.

PS concentrations were below the LOQ at both sites, a common finding in wastewater studies showing PS degradation nor removal during preliminary treatment stages. Its rapid breakdown and lower stability in wastewater systems often result in concentrations below DL.

In comparisonto other results,^11^ influent PE concentrations in other WWTPs were reported at 2340, 1481, and 607 μg/L — 5 to 20 times higher than Avedøre samples but comparable to Pontedera. PP levels in Avedøre were below Okoffo’s LOQ of 8 μg/L.

These findings align with current wastewater research, demonstrating the influence of site-specific factors, influent wastewater composition, and operational differences in WWTPs on MP concentrations. The observed variability highlights the need for customized MP quantification protocols to accurately track MP distribution and behavior in wastewater systems.

Moreover, a detailed comparison between the quantified MP ratios and their expected confidence intervals is provided in the Supporting Information (Table S10). While most samples exhibited ratios consistent with expected ranges, PET and PP presented notable deviations, particularly in lake samples (A, B, and C). These discrepancies may be attributed to polymer degradation,^39^ environmental accumulation patterns,^40,41^ or matrix interferences affecting quantification.^30^ Given the inherent variability in MP composition and the influence of additives and polymer aging, further refinement of calibration strategies and extraction protocols could enhance quantification accuracy. However, the overall consistency in PE and PS detection, along with the observed trends in wastewater and surface water samples, supports the robustness of the analytical approach applied in this study.

## Conclusions

This study established a refined approach for isolating MPs—PE, PET, PP, and PS—in environmental and wastewater samples using double-shot pyrolysis-gas chromatography/mass spectrometry, with or without PLE. The method effectively addressed matrix interferences, by either thermal desorption to remove volatile and semivolatile compounds before pyrolysis and/or by pre-extraction with methanol using PLE.

Key findings provide valuable insights into critical parameters influencing MP quantification. The selected extraction strategy, whether using PLE with methanol pre-extraction followed by tetrahydrofuran or direct analysis via Py-GC/MS will reduce quantification biases and improve MP recovery. The final pyrolysis conditions, set at 625 °C for 40 s, delivered consistent sensitivity and reproducibility. The study also evaluated different quantification strategies, showing that solid MP calibration curves improved accuracy for PET and PP, while solubilized calibrations provided better linearity for PE and PS.

Tests of surface water from a lake in Copenhagen and wastewater from two sites in Italy and Denmark showed that PET and PE were the most abundant MPs, likely due to their stability and density, whereas PP and PS were detected at lower levels, influenced by their buoyancy and degradation potential. Variations observed between WWTPs showed the impact of influent composition and operational factors on MP concentrations.

Further efforts should focus on refining Py-GC/MS conditions and evaluating the necessity of PLE for different sample types to enhance detection limits and overall performance. Developing consistent calibration protocols to ensure comparability across laboratories and sample types – the study clearly showed the challenge with obtaining reliable calibration solution either solid or liquid.

This study presents a analytical strategy for MP monitoring, offering flexible approaches for effective extraction, pyrolysis, and quantification to support environmental assessments and regulatory initiatives.

## Supplementary Material



## Notes

General experimental methods; materials and reagents, synthesis; characterizations; calibration data, additional figures, and spectra (PDF)
