# Supplementary material for: Quantification Challenges in Polymer Analysis in Urban Runoff and Wastewater using Pressurized Liquid Extraction and Double-Shot Pyrolysis-Gas Chromatography-Mass Spectrometry
Source: Anal Chem. 2025 Jul 7;97(27):14321–30. doi: 10.1021/acs.analchem.5c01170 (PMC12268836; doi:10.1021/acs.analchem.5c01170)
Supplement: Supplementary file 1 [file ac5c01170_si_001.pdf]

Supporting Information

for

Quantification Challenges in Polymer Analysis in Urban Runoff and Wastewater using Pressurized Liquid Extraction and Double-Shot Pyrolysis-Gas Chromatography-Mass Spectrometry

Daniele Martuscelli*[1,2], Jonas B. Jensen[2], Luca Solari[1], Simona Francalanci[1], Peter Christensen[2], Jan H. Christensen[2].

[1] · Department of Civil and Environmental Engineering, University of Florence

[2] Analytical Chemistry Group, Department of Plant and Environmental Science, Faculty of Science, University of Copenhagen, Thorvaldsensvej 40, 1871 Frederiksberg, Denmark.

*Corrisponding author. E-mail address: daniele.martuscelli@unifi.it (D.Martuscelli)

Table of Contents

	Contents	Page
**SI1**	Sampling sites and preparation	S-3
**SI2**	Materials	S-3
**SI3**	Chemicals	S-3
**SI4**	Preparation of MP materials	S-4
**SI5**	Polymer solutions in pyrovials for preparation of calibration curves	S-4
**SI6**	Solubilized MP Calibration Curves	S-4
**SI7**	Solid Particle MP Calibration Curves	S-4
**SI8**	Polymer solubility and handling challenges	S-4
**SI9**	Effect of loading volume on pyro-vial response signal	S-5
**SI10**	Pressurized liquid extraction	S-5
**SI11**	DL and LOQ determinations	S-5
**Table S1**	Comparison of selected ion peak areas, normalized to the 1:50 split ratio.	S-6
**Table S2**	Effect of TDU hold time on carry over.	S-7
**Table S3**	Linear regression parameters for calibration curves of PE, PET, PP, and PS with 4-fluorostyrene (*m/z*=96) as internal standard	S-8
**Table S4**	Quantifier-to-Qualifier Ratios for PE, PET, PP, and PS in different environmental samples.	S-9
**Table S5**	Overview of scan windows used.	S-10
**Table S6**	An example of a calibration curve batch design where the samples range from 200 ng to 10 ug.	S-11
**Table S7**	PE, PET, PP, and PS concentration in water samples A, B, and C (n=4) from Utterslev Mose	S-12
**Table S8**	An example of a calibration curve batch design	S-13
**Table S9**	PE, PET, PP, and PS concentration in water samples A, B, and C from Utterslev Mose, along with MilliQ and tap water blank samples	S-14
**Table S10**	Ratios of the peak area	S-15
**Figure S1**	Location of WWTP water sample (43°39'48.8"N 10°36'53.4"E) at Pontedera (PI) Italy	S-16
**Figure S2**	Location of water sample A (55°42'56.9"N 12°30'09.3"E) and water samples B and C (55°43'07.8"N 12°30'46.8" E) at Utterslev Mose.	S-16
**Figure S3**	Comparison of the quantification of polymer PS present in the samples with and without homogenization phase	S-17
**Figure S4**	Images of dried pyro-vials	S-18
**Figure S5**	Overlayed chromatogram peaks for PS pyrolysis with different inorganic matrices.	S-19
**Figure S6**	Linear calibration curves, data points, and % error plots for selected analytes	S-20 - S-21
**Figure S7**	TIC’s from TDU cleaning process	S-22
**Figure S8:**	EIC’s of *m/z*=111, being the quantifier ion for the PP pentamer, in top row and *m/z*=210, qualifier ion, in bottom row.	S-23
**Figure S9**	EIC’s of *m/z*=111, being the quantifier ion for the PP pentamer	S-24

SI1 - Sampling sites and preparation

The PLE-Py/GC-MS method was applied to wastewater samples from two WWTPs: Pontedera (Italy), where the municipal spillway receives residential, road runoff, and industrial wastewater (Figure SI1), and Avedøre (Denmark), where effluent is discharged after treatment. During heavy rainfall, the Pontedera spillway activates per Italian regulations. Samples were filtered to isolate MPs.

Additionally, surface water was collected from three sites (A, B, C) in Utterslev Mose (Denmark), with site C located under a bridge with traffic (Figure SI2). At each site, 2.5 L of water was sampled in glass bottles and refrigerated until analysis.

Samples from Pontedera and Avedøre WWTPs were vacuum-filtered (0.7 µm), requiring multiple rounds due to clogging. The retained material was analyzed for MPs. Before PLE-Py/GC-MS, samples were extracted using PLE, with dried filters folded, placed in 33 mL ASE-200 cells, and packed with Ottawa sand (1 cm from the top), following the Section 2.8 protocol.

Utterslev Mose samples were sequentially filtered (1.6 µm and 0.7 µm, Whatman GF/A) to match control spike conditions. MilliQ and tap water controls were processed alongside. Filters were dried (105 °C, 4 h) and cryo-milled, with both filter sizes combined before milling.

SI2 - Materials

The plastic polymers used were: “Flexirene” linear low-density polypropylene (LLDPE or PE for simplicity, Versalis, pellets), “PP1013H1” polypropylene homopolymer (PP, Exxonmobile, pellets), “Eastar copolyester MN058” polyethylene terephthalate (PET, Eastman, pellets), “Styron 485” polystyrene (PS, Trinseo, pellets), Poly(4-fluorostyrene) (fluoro-PS, sample #: P43494-4FS, Polymer Source). The MP materials were provided by CC Plast in Hillerød, Denmark. The MP materials were in pellet form, each a few millimeters in diameter.

Quartz glass pyrolysis vials, with no slit (length 17 mm, part No. 018133-100-00, Gerstel), were used as sample containers during pyrolysis. Tools and containers made of plastic materials were avoided during the handling and preparation of plastic polymer solutions. Glass- and metalware was used instead. The only plastic material in contact with the reagents during sample preparation and analysis was PTFE-vial caps, used to close the 8 mL glass vials for polymer solubilization

SI3 - Chemicals

The solvents used in this study were chloroform (ACS grade, ≥99.8%, Sigma-Aldrich), toluene (glass distilled grade, Rathburn), dichloromethane (glass distilled grade, Rathburn), 1-octanol (Reidel-de Haën), trifluoroacetic acid (TFA, ≥99%, Sigma-Aldrich), 1,2,4-trichlorobenzene (TCB, ≥99%, Sigma-Aldrich), and tetrahydrofuran (THF, ACS reagent, with BHT inhibitor, ≥99%, Sigma-Aldrich).

These chemicals are volatile and toxic; all handling was performed in a fume hood with appropriate PPE.

SI4 - Preparation of MP materials

MP sample preparation involved cryo-milling the pellets (few mm size) into fine powder. 1–2 g of polymer were placed in stainless-steel grinding jars with a 20 mm steel ball, submerged in liquid nitrogen (10 min, twice), and milled using a TissueLyser II (30 Hz, 3–10 min, QIAGEN). For tougher polymers (e.g., PE), milling cycles were repeated with additional cooling.

The powder was sieved (100, 250, 500 µm), repeating the process as needed to collect 100–200 µm particles. Only >100 µm particles were used, aligning with environmental survey thresholds[1–3].

SI5 - Polymer solutions in pyrovials for preparation of calibration curves

Solubilized polymer solutions were prepared by dissolving cryo-milled MPs in 8 mL glass vials with selected solvents (20:80 octanol for PE/PP; 30:70 TFA for PET/PS). Solutions were heated to dissolve MPs (40°C for PET/PS, 130°C for PE/PP), with volatile solvents added first and evaporated at 100°C overnight (≥8 h).

MP solutions were loaded into pyro-vials using climatized syringes (50 µL for MPs, 10 µL for fluoro-PS), starting with lower MP concentrations to avoid contamination. Syringes were equilibrated by flushing three times before use and cleaned in heated octanol (130°C) using a two-step rinse process.

SI6 - Solubilized MP Calibration Curves

Stock solutions of PE/PP (octanol 20:80) and PET/PS (TFA 30:70) were prepared at 0.9 µg/µL (I), 0.3 µg/µL (II), and diluted to 35 ng/µL (III). A six-point calibration curve (200 ng–10 µg, n = 3) was constructed.

Samples were analyzed in three batches, with pyrolysis vial adaptors pre-cleaned at 750°C (40 s). Cleaning steps included TDU at 350°C (30 min) and CIS at 300°C (25 min). To reduce carryover, samples were run low to high concentration, with blank vials in between.

SI7 - Solid Particle MP Calibration Curves

A six point calibration curve was created using solid MP particles (PE, PET, PP and PS) mixed with Hydromatrix. The MP particles were cryo-milled, sieved to >100 µm, and combined with Hydromatrix to achieve pyro-vial loadings of 200 ng, 400 ng, 1 µg, 2 µg, 6 µg, and 10 µg, with two replicates per point (n = 2). Each pyro-vial was spiked with 0.95 µg fluoro-PS (solubilized in THF), dried, and filled with 2-3 mg of the Hydromatrix-MP mix.

SI8 - Polymer solubility and handling challenges

PS was the easiest polymer to dissolve due to its large interaction radius (Ro), dissolving in most tested solvents except TFA and octanol (high δH values, Table 1), consistent with studies using DCM:THF (50:50)[4] and TCB at 120°C[5]. PET dissolved in 70:30 Chloroform:TFA (RED = 0.92) at room temperature, while DCM (RED = 0.99) required high temperature and pressure[6]. PE and PP were harder to dissolve; 20:80 octanol:toluene was most effective for PE, but both required heating

(100°C for PE, 130°C for PP), suggesting HSP spheres do not fully predict solubility at high temperatures[7]. Attempts with TCB[5] failed due to density differences, causing polymers to float and precipitate upon cooling, in line with Matsueda et al. (2021). PE and PP also presented handling issues, solidifying on vial surfaces upon repeated heating, leading to concentration loss. Stock solutions were prepared in larger volumes to minimize surface exposure, and heated syringes (50 µL, larger inner diameter) were used to prevent clogging. In contrast, PET and PS in chloroform allowed precise aliquoting with smaller syringes.

SI9 - Effect of loading volume on pyro-vial response signal

The impact of loading volume on response signal was evaluated by loading a 20 µL PP solution using three methods: a single 20 µL load, two sequential 10 µL loads with evaporation in between, and four sequential 5 µL loads with evaporation. A 50 µL syringe was used, with a 1:100 split ratio and a 180 mL/min TDU flow during pyrolysis (MS scan mode: 10-500 m/z).

Incremental loading of smaller volumes resulted in a slightly higher detector signal for the PP trimer, likely due to more concentrated deposition at the vial bottom. A similar trend was observed for the two larger PP pentamer peaks. Visual inspection confirmed that smaller volumes concentrated PP at the vial base, while larger volumes caused distribution further up the pyro-vial. When adding the volume as 4 × 5 µL method, a thin PP layer formed along the inner vial walls, likely due to surface tension between the syringe needle and vial wall, creating a “curtain” effect (Figure SI5).

SI10 - Pressurized liquid extraction

PLE was used to isolate MPs while minimizing interference. Samples were filtered (GB-140, 7 µm), dried, folded, and placed in 33 mL ASE-200 cells packed with Ottawa sand (1 cm from the top).

A two-step PLE method was optimized: a 100°C methanol pre-extraction to remove micropollutants, followed by THF extraction at 180°C. Key parameters: 1500 psi, 5 min static time, 3 methanol cycles, 2 THF cycles, flush volumes (MeOH 45%, THF 80%), 75 s purge. Extracts were collected in 80 mL vials with preheated Hydromatrix (450°C).

PLE efficiency was assessed via hydrothermal extraction (180°C, 16 h, 1°C/min increase) and by weighing vials before/after solvent evaporation. Two methods were tested: (1) three rinses (50% volume, combined) and (2) one rinse (150% volume). A four-cycle extraction was also evaluated for full polymer recovery

SI11 - DL and LOQ determinations

DL and LOQ were determined by calculating the standard deviation of 23 blank samples, adding the mean blank signal for each pyrolyzate (Table SI2), and dividing by the slope of the calibration curve, as shown in equations 1 and 2:

$$DL = \frac{(3\sigma + \text{Mean of blanks})}{m} \quad (1)$$

$$LOQ = \frac{(10\sigma + \text{Mean of blanks})}{m} \quad (2)$$

Where σ represents the standard deviation of the blank samples (n=23), and m is the slope of the calibration curve.

Table S1 Dispersive (δD), polar (δP), and hydrogen-bonding (δH) parameters for microplastics (MPs) and selected solvents, along with the interaction radius (Ro) for each MP.

	δ_D	δ_P	δ_H	R_o
**Polymers**				
**PE**	16.0	0.8	2.8	3.2
**PET**	19.1	6.3	9.1	4.8
**PP**	18.0	0.0	1.0	6.0
**PS**	22.3	5.8	4.3	12.7
**Solvents**				
**TFA**	15.6	9.7	11.4	
**DCM**	17.0	7.3	7.1	
**THF**	16.8	5.7	8.0	
**TCB**	20.2	4.2	3.2	
**Toluene**	18.0	1.4	2.0	
**Chloroform**	17.8	3.1	5.7	
**Octanol**	16.0	6.0	11.2	

Table S2: Plastic polymers, target pyrolyzates, and corresponding m/z ions for peak integration with quantifier and qualifier ions used for peak integration are listed, where bolded m/z values represent ions used for quantification.

Polymer	Indicator Compound(s)	Indicator Fragments (m/z)	
		Qualifier ions	Quantifying ion
**PS**	Styrene	51, 104	**78**
	3-buten-1,3-diyldibenzene (styrene dimer)	91,130	208
	5-hexen-1,3,5-triyltribenzene (styrene trimer)	117, 194, 207, 312	91
**PET**	Benzopehone	77, 105, 152	**182**
	Dimethyl terephthalate	76, 104	175
	Vinyl benzoate	51, 77	105
	Biphenyl	76,152	154
**PP**	2,4-dimethyl-1-hept-1-ene	43, 55, 70	**126**
	2,4,6,8-tetramethyl-1-undecene	43, 69	111
**PE**	1-Decene (C_{10})	83, 140	**97**
	1-Undecene (C_{11})	82, 96, 112	154
	1-Tridecene (C_{13})	96, 112, 154, 182	196
	1-Tetradecene (C_{14})	69, 97	83

Table S3: Comparison of selected ion peak areas, normalized to the 1:50 split ratio, where Pyrolyzate names are listed under each MP with extracted ion m/z values in brackets.

		Normalized peak area to 1:50			
**Pyrolyzate**	**Split ratio:**	**1:50**	**1:25**	**1:10**	**Splitless**
**PE**	**RI**				
1-Decene (97)	992	1	1.3	1.9	1.5
1-Undecene (97)	1092	1	1.2	1.5	1.7
1-Dodecene (97)	1185	1	1.1	1.7	1.7
1-Tridecene (97)	1292	1	1.2	1.6	1.2
1-Tetradecene (97)	1393	1	1.2	1.4	4.1
**PET**					
Vinyl Benzoate (148)	1137	1	0.9	0.8	6.4
Biphenyl (154)	1393	1	0.8	1.0	7.1
Divinyl terepthalate (104)	1577	1	1.9	3.1	24.5
Benzophenone (182)	1650	1	1.0	1.8	26.8
**PP**					
PP - Trimer (126)	842	1	1.4	1.5	1.3
PP - Pentamer 1 (111)	1309	1	1.4	1.5	1.6
PP - Pentamer SUM (111)		1	1.3	1.4	1.6
**PS**					
Styrene (77)	891	1	1.3	1.4	1.3
PS - Dimer (91)	1740	1	2.2	4.1	22.4
2,5-Diphenyl-1,5-hexadiene (234)	1915	1	3.3	11.8	33.3
PS - Trimer (91)	2480	1	4.5	19.1	25.8

Table S4: Effect of TDU hold time on carryover showing retention time changes and carryover percentages for 1-min and 5-min holds after pyrolysis

	Retention time [*min*]			Carryover %			
**TDU hold:**	**1 min**	**5 min**	**Δ Rt**	**1 min**	**5 min**	**1 min**	**5 min**
**PE**							
1-Decene	4.968	4.681	0.29	2.9	1.1	2.3	2.6
1-Undecene	6.110	5.996	0.11	2.9	0.9	2.2	2.2
1-Dodecene	7.151	7.106	0.04	3.1	1.0	2.3	1.7
1-Tridecene	8.125	8.107	0.02	2.7	0.9	1.8	1.5
1-Tetradecene	9.030	9.025	0.00	2.5	0.9	2.4	1.3
**PET**							
Vinyl Benzoate	6.624	6.537	0.09	0.9	0.6	1.0	1.0
Biphenyl	9.034	9.021	0.01	8.8	6.4	9.2	6.5
Divinyl terepthalate	10.540	10.540	0.00	5.5	0.9	6.4	1.8
Benzophenone	11.113	11.113	0.00	13.7	8.3	17.9	8.5
**PP**							
PP - Trimer	3.030	1.770	1.26	1.7	0.4	1.1	1.2
PP - Pentamers							
Peak 1	8.279	8.261	0.02	1.2	0.3	0.8	0.6
Peak 2	8.352	8.339	0.01	2.3	1.1	1.4	1.2
Peak 3	8.434	8.420	0.01	1.0	0.3	0.7	0.5
**PS**							
Styrene	3.731	2.929	0.80	0.3	0.1	0.2	0.2
PS - Dimer	11.786	11.786	0.00	9.0	1.9	11.4	3.3
2,5-Diphenyl-1,5-hexadiene	13.010	13.010	0.00	10.4	2.7	18.8	4.2
PS - Trimer	16.344	16.344	0.00	69.0	7.3	123.5	10.0

Table S5: Linear regression parameters for calibration curves of PE, PET, PP, and PS using 4-fluorostyrene (m/z=96) as an internal standard, with y as the analyte-to-internal standard peak area ratio and x as the analyte-to-internal standard concentration ratio

	Solution - IS		Solids - IS	
**PE**	**Intercept**	**Slope**	**Intercept**	**Slope**
**1-Decene (97)**	-4.1E-04	5.3E-03	4.1E-04	6.7E-03
**1-Undecene (97)**	-7.8E-04	4.0E-03	-3.3E-05	4.4E-03
**1-Undecene (154)**	-7.5E-05	4.7E-04	2.4E-05	4.9E-04
**1-Dodecene (97)**	-2.9E-04	4.8E-03	9.5E-04	4.6E-03
**1-Tridecene (97)**	-5.6E-04	4.4E-03	3.0E-04	5.3E-03
**1-Tetradecene (97)**	6.7E-05	5.5E-03	2.3E-03	6.9E-03
**PET**				
**Biphenyl (182)**	6.5E-03	3.4E-02	1.2E-02	9.9E-02
**Benzopehone (182)**	-9.5E-05	8.2E-03	-9.0E-04	2.7E-02
**PP**				
**Trimer (126)**	-3.7E-03	2.0E-02	-1.6E-03	2.8E-02
**Pentamer 1 (111)**	-1.3E-03	7.6E-03	-8.1E-04	1.0E-02
**Pentamer SUM (111)**	-2.4E-03	1.4E-02	-1.7E-03	1.9E-02
**PS**				
**Styrene (78)**	2.1E-01	8.6E-01	-8.5E-03	7.6E-01
**Styrene SUM (77+78)**	3.2E-01	1.3E+00	1.6E-02	1.1E+00
**2,5-Diphenyl-1,5-hexadiene (234)**	-1.6E-03	8.0E-03	-1.2E-03	4.0E-03

Table S6: Quantifier-to-Qualifier ratios for PE, PET, PP, and PS in environmental samples representing the ratio of the quantifier peak to the qualifier peak for each polymer

	Ratio			
Sample	**PE**	**PET**	**PP**	**PS**
Alba	3,35	0,44	2,05	181,38
Ponte	3,50	0,45	2,36	550,96
Sample A	3,53	3,91	0,42	274,44
Sample B	3,00	1,84	0,93	247,04
Sample C	3,19	1,10	0,00	530,24
MilliQ	5,22	0,01	2,66	0,58
Tap Water	3,45	0,02	2,31	0,99

Table S7: Overview of scan windows used, including target microplastic polymer, pyrolytic target, scan start time (min), and ion masses with high resolution, six unique ions per window (30 ms dwell time), and adjustments excluding vinyl benzoate while including PP pentamer peaks.

Target MP	Name	Time (min)	*m/z*
PP	2,4-Dimethyl-1-heptene	0	126
PS	Styrene		104, 77, 78
IS-PS	Fluoro- Styrene		122, 96
PS	α-Methylstyrene	4.4	118, 103
PE - C10	1-Decene		140, 97
PE - C10	1,9-Decadiene		110, 81
PET	Acetophenone	5.4	120, 105
PE - C11	1-Undecene		154, 97
PE - C11	1,10-Undecadiene		124, 81
PET	Vinyl Benzoate	6.5	105, 148
PE - C12	1-Dodecene	7.5	168, 97
PE - C12	1,11-Dodecadiene		81
PE - C13	1-Tridecene		182, 97
PE - C13	1,12-Tridecadiene		81
PP	Pentamer		210, 111
PET	Biphenyl	8.7	154, 76
PE - C14	1-Tetradecene C14		196, 97
PE - C14	1,13-Tetradecadiene		194, 81
PS	Bibenzyl	9.6	182, 91
PET	Divinyl terephthalate		175, 154, 105
PET	Benzophenone		182, 105
PS	Dimer	11.3	208, 130, 91
IS-PS	Dimer - fluoro		229, 133
PS	2,5-Diphenyl-1,5-hexadiene		234, 130
PS	Trimer	13.4	312, 207, 91
PET	2-(benzoyloxy)ethyl vinyl terephthalate		297, 149, 105

Table S8: Example of a calibration curve batch design with samples from 200 ng to 10 µg, including exact MP polymer weights per vial, cleaning steps, blanks, internal standard (fluoro-PS), and calibration samples with notes on MP solutions and volumes used.

		**Solvent**	**Octanol:Toluene (20:80)**		**TFA:Chloroform (30:70)**		**Octanol:Toluene (20:80)**		**TFA:Chloroform (30:70)**		**THF**		
		**Stocks 1**	0.90 *µg/µL*		0.86 *µg/µL*		0.90 *µg/µL*		0.87 *µg/µL*		0.32 *µg/µL*		
		**Stocks 2**	0.30 *µg/µL*		0.31 *µg/µL*		0.34 *µg/µL*		0.28 *µg/µL*				
		**Dilutions 3**	34.12 *ng/µL*		35.10 *ng/µL*		39.02 *ng/µL*		32.49 *ng/µL*				
**Place #**	**Run #**	**Name**	**PE**		**PET**		**PP**		**PS**		**Fluoro-PS**		**Note**
			µL		***µL***		***µL***		***µL***		***µL***	***µg***	
1	1	**Bake 1**											
1	2	**Blank 1**											
2	3	**Syringe cleaning**	10										
1	4	**Blank 2**											
3	5	**SIM IS-PS Only**									3	0.95	
1	6	**Blank 3**											
4	7	**SIM 200ng 1**	6	205 ng	6	211 ng	6	234 ng	6	195 ng	3	0.95	Dilutions 3
1	8	**Blank 4**											
5	9	**SIM 400ng 1**	12	409 ng	12	421 ng	11	429 ng	12	390 ng	3	0.95	Dilutions 3
1	10	**Blank 5**											
6	11	**SIM 1ug 1**	4	1.2 µg	4	1.2 µg	4	1.4 µg	4	1.1 µg	3	0.95	Stocks 2
1	12	**Blank 6**											
7	13	**SIM 2ug 1**	7	2.1 µg	7	2.2 µg	7	2.4 µg	7	2.0 µg	3	0.95	Stocks 2
1	14	**Blank 7**											
8	15	**SIM 6ug 1**	7	6.3 µg	7	6.0 µg	7	6.3 µg	7	6.1 µg	3	0.95	Stocks 1
1	16	**Blank 8**											
9	17	**SIM 10ug 1**	12	10.8 µg	12	10.3 µg	12	10.8 µg	12	10.4 µg	3	0.95	Stocks 1
1	18	**Blank 9**											
1	19	**Bake 2**											
1	20	**Blank 10**											

Table S9: PE, PET, PP, and PS concentrations in water samples A, B, and C from Utterslev Mose, MilliQ, and tap water blanks, quantified using solubilized MP calibration with fluorostyrene (m/z=96) as an internal standard, showing mean concentration and 95% confidence interval in µg/L with superscripts indicating values below DL, LOQ, or above the calibration range.

Pyrolyzate (*m/z*)	Sample A [µg/L]		Sample B [µg/L]		Sample C [µg/L]		MilliQ [µg/L]		Tap water [µg/L]	
	Mean	± CI(95)	Mean	± CI(95)	Mean	± CI(95)	Mean	± CI(95)	Mean	± CI(95)
**PE**										
1-Decene (97)	81.7	± 32.1	158.4	± 68.6	268.1	± 143.9	10.5	± 2.2	9.3	± 3.7
1-Undecene (97)	111.4	± 79.8	322.3	± 108.9	2816.7[c]	± 1297.6	13.3	± 1.0	13.1	± 4.3
1-Undecene (154)	101.8	± 41.9	237.6	± 108.2	398.4	± 182.3	12.5[a]	± 1.6	14.6[a]	± 4.7
1-Dodecene (97)	90.8	± 27.6	187.4	± 159.0	630.4[c]	± 81.2	11.2[b]	± 2.3	11.2[b]	± 4.7
1-Tridecene (97)	134.3	± 29.2	363.1	± 126.4	291.6	± 111.4	12.0[b]	± 1.6	11.8[b]	± 3.7
1-Tetradecene (97)	190.0	± 47.3	195.1	± 41.9	363.2	± 155.5	18.5[a]	± 4.3	16.3[a]	± 6.7
**PET**										
Biphenyl (182)	-1.9[a]	± 1.0	9.6[a]	± 18.7	33.2[b]	± 62.5	5.6[a]	± 8.8	-2.8[a]	± 0.7
Benzopehone (182)	91.6	± 18.6	133.5	± 27.1	195.0	± 94.1	1.2[a]	± 0.9	0.8[a]	± 0.2
**PP**										
PP - Trimer (126)	8.3	± 0.7	8.2	± 0.4	10.6	± 1.6	8.6331	± 1.1	7.8	± 0.4
PP - Pentamer peak 1 (111)	14.2	± 5.9	9.4	± 1.5	1219.4[c]	± 397.8	8.4631	± 1.0	7.8	± 0.5
PP - Pentamer SUM (111)	13.0	± 3.3	11.1	± 1.2	1348.8[c]	± 440.0	8.4237	± 0.9	7.7	± 0.5
**PS**										
Styrene (78)	-7.3[a]	± 1.3	-4.2[a]	± 2.5	-2.1[a]	± 5.0	-9.4[a]	± 0.0	-9.4[a]	± 0.0
Styrene SUM (77+78)	-7.8[a]	± 1.4	-4.5[a]	± 2.7	-2.4[a]	± 5.3	-9.9[a]	± 0.0	-9.9[a]	± 0.0
2,5-Diphenyl-1,5-hexadiene (234)	8.8[b]	± 0.3	10.6[b]	± 1.5	12.2[b]	± 1.5	8.2[b]	± 0.9	7.8	± 0.1

Table S10: Ratios of the peak area of the qualifier compound to the peak area of the quantifier compound for the quantification of microplastics in water samples (*values outside of the confidence interval).

	Ratio qualifier				
**Pyrolyzate (m/z)**	**Sample A**	**Sample B**	**Sample C**	**MilliQ**	**Tap water**
**PE**					
1-Decene (97)	1.0	1.0	1.0	1.0	1.0
1-Undecene (154)	0.8	0.7	0.7	1.1	0.7
1-Tridecene (97)	0.6	0.4	0.9	1.1	0.9
1-Tetradecene (97)	0.5	0.9	0.8	0.5	0.4
**PET**					
Benzopehone (182)	1.0	8.1	4.8	0.1	0.1
Biphenyl (182)	16.8	1.0	1.0	1.0	1.0
**PP**					
2,4-dimethyl -1-hept-1-ene (126)	1.0	1.0	1.0	1.0	1.0
2,4,6,8-tetramethyl-1-undecene (111)	0.2	0.2	0.0	1.0	0.8
**PS**					
Styrene (78)	1.0	1.0	1.0	1.0	1.0
2,5-Diphenyl-1,5-hexadiene (234)	1.6	1.4	3.1	0.0	0.0

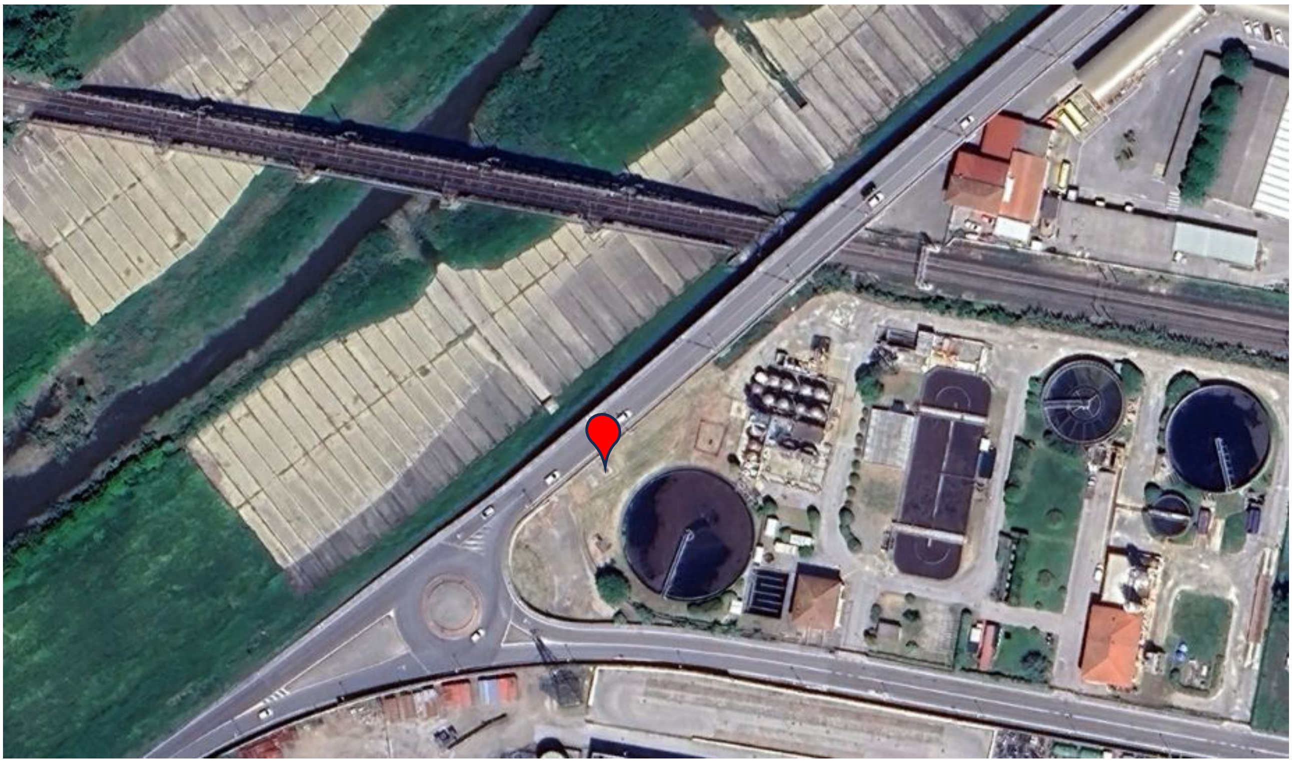

Figure S1*:* Location of WWTP water sample (43°39'48.8"N 10°36'53.4"E) at Pontedera (PI) Italy

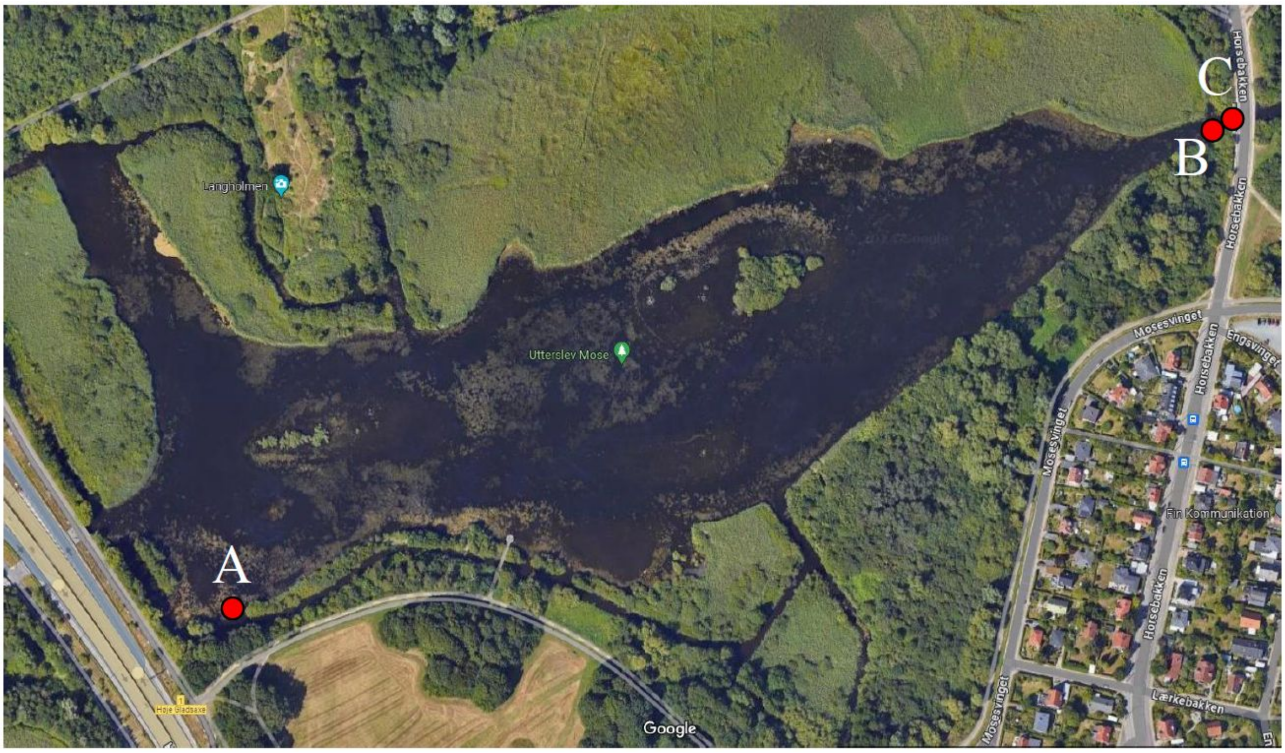

Figure S2: Location of water sample A (55°42'56.9"N 12°30'09.3"E) and water samples B and C (55°43'07.8"N 12°30'46.8" E) at Utterslev Mose.

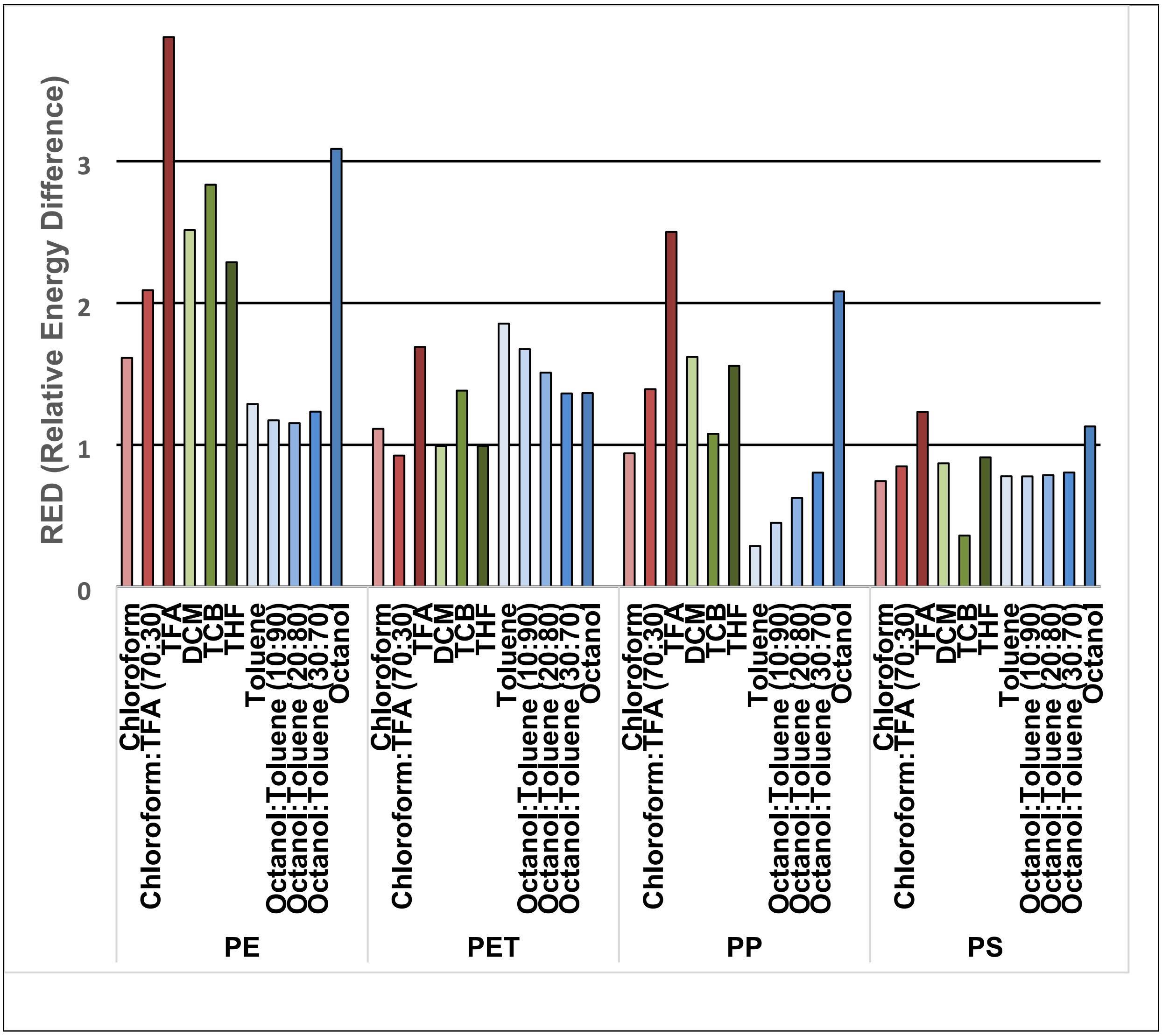

Figure S3: RED values (y-axis) for seven solvents (chloroform, TFA, DCM, TCB, THF, toluene, and octanol) and four solvent mixtures (one chloroform and three octanol-based) evaluated for the four polymers: PE, PET, PP, and PS (x-axis).

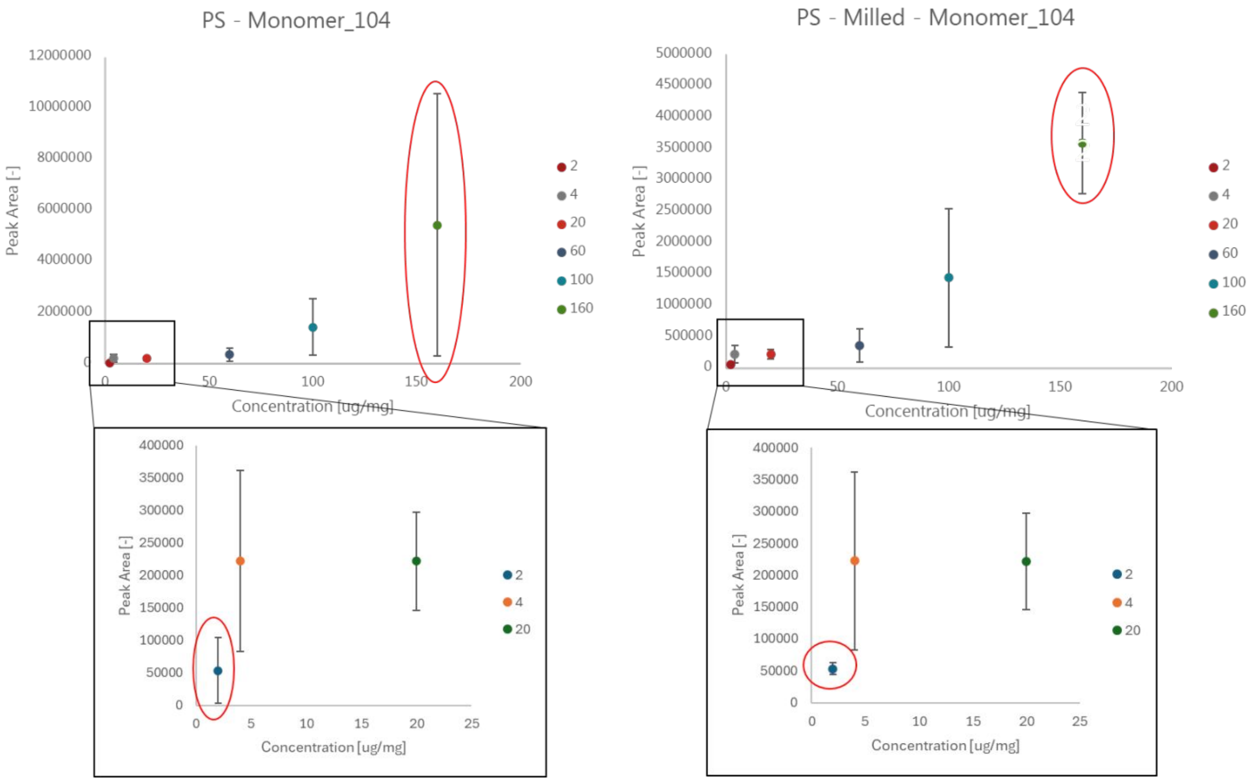

Figure S4: Comparison of the quantification of polymer PS present in the samples with and without homogenization phase

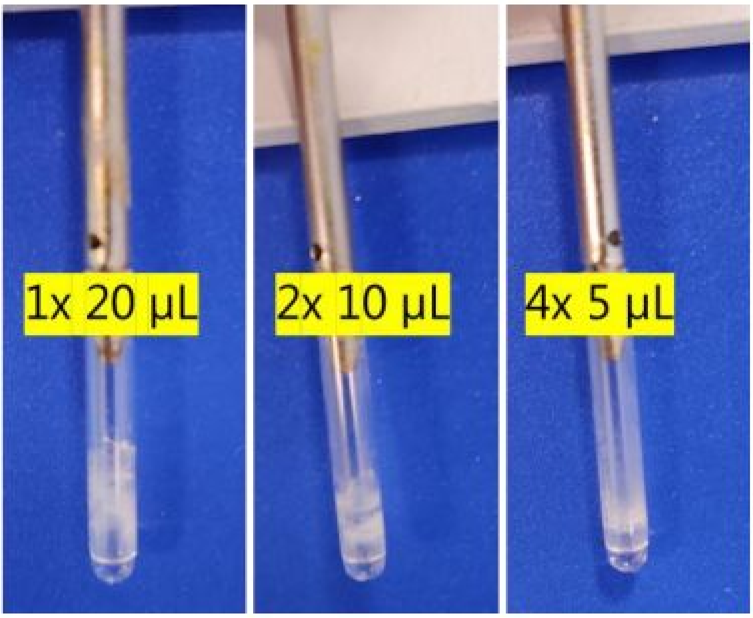

Figure S5: Images of dried pyro-vials loaded with 20 µL of PP solution in 1, 2, or 4 injections.

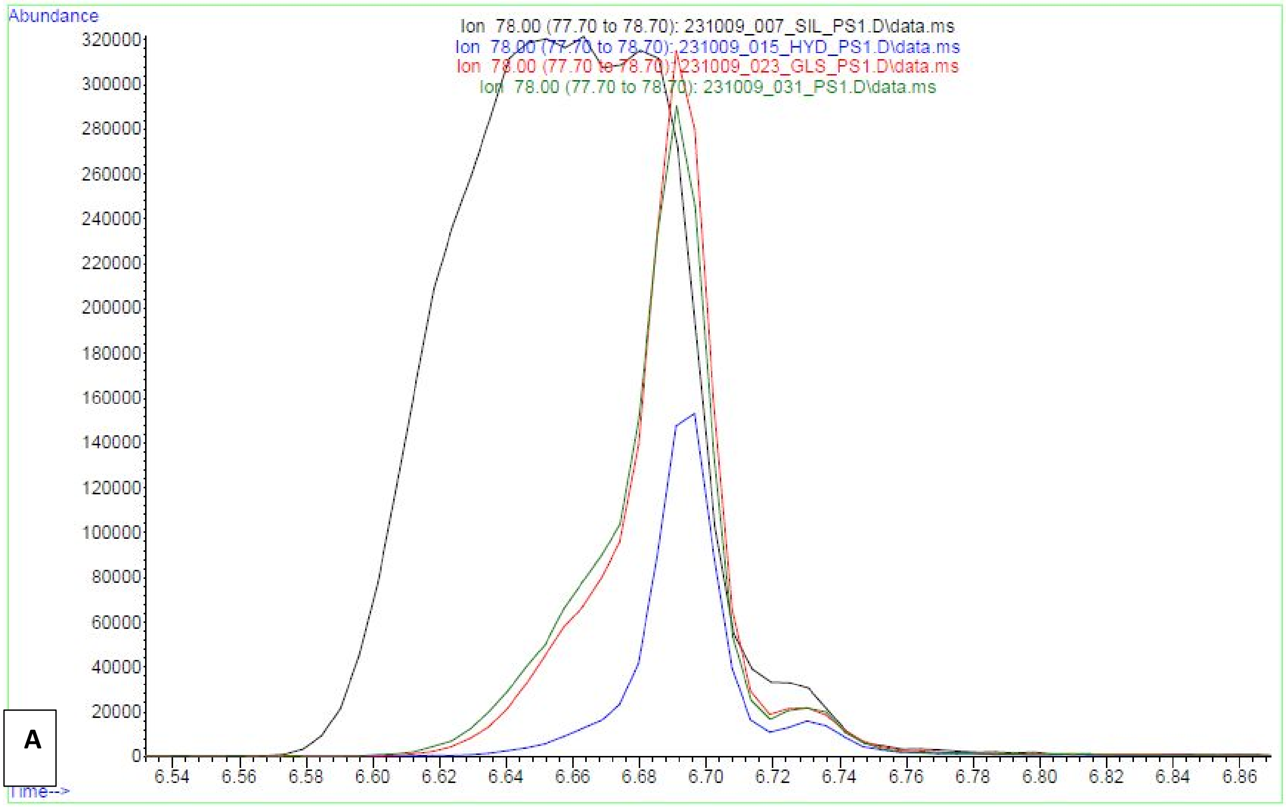
A
Abundance
Ion 78.00 (77.70 to 78.70): 231009_007_SIL_PS1.D\data.ms
Ion 78.00 (77.70 to 78.70): 231009_015_HYD_PS1.D\data.ms
Ion 78.00 (77.70 to 78.70): 231009_023_GLS_PS1.D\data.ms
Ion 78.00 (77.70 to 78.70): 231009_031_PS1.D\data.ms
Time-->

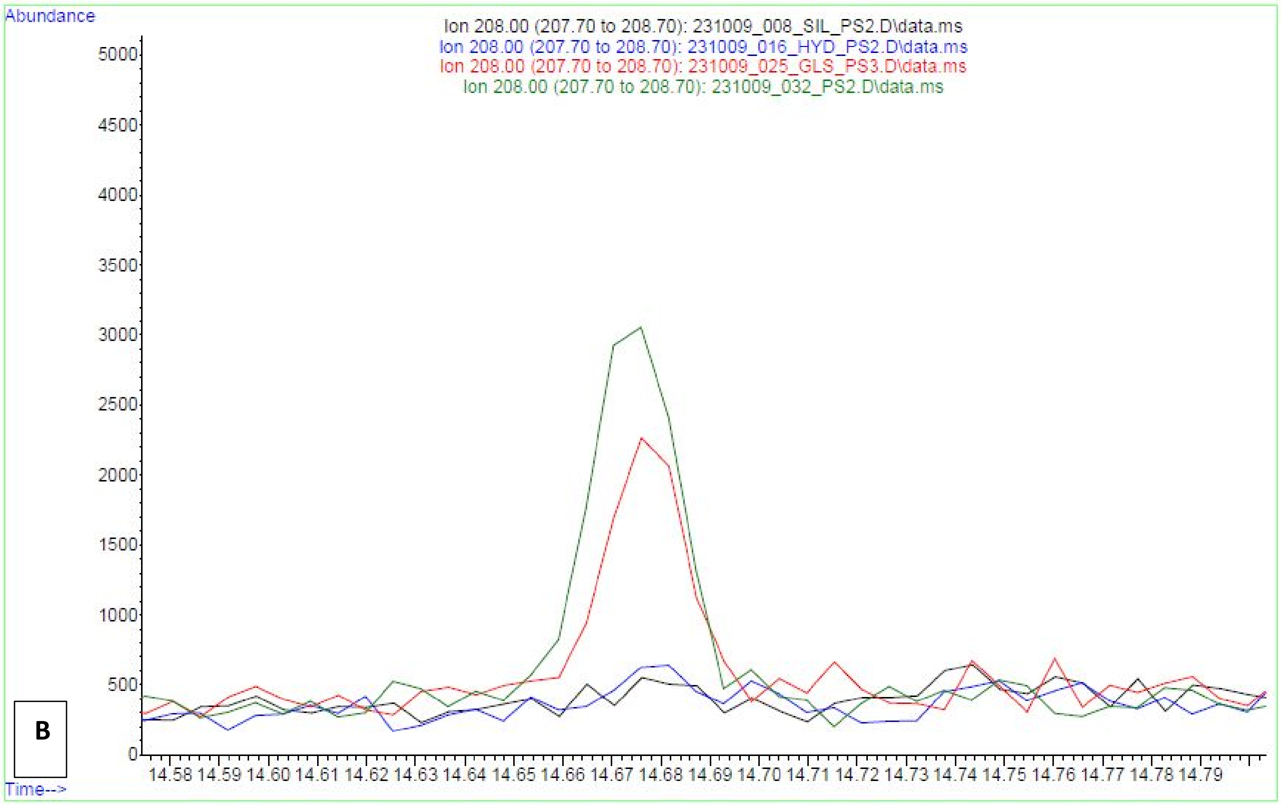
B
Abundance
Ion 208.00 (207.70 to 208.70): 231009_008_SIL_PS2.D\data.ms
Ion 208.00 (207.70 to 208.70): 231009_016_HYD_PS2.D\data.ms
Ion 208.00 (207.70 to 208.70): 231009_025_GLS_PS3.D\data.ms
Ion 208.00 (207.70 to 208.70): 231009_032_PS2.D\data.ms
Time-->

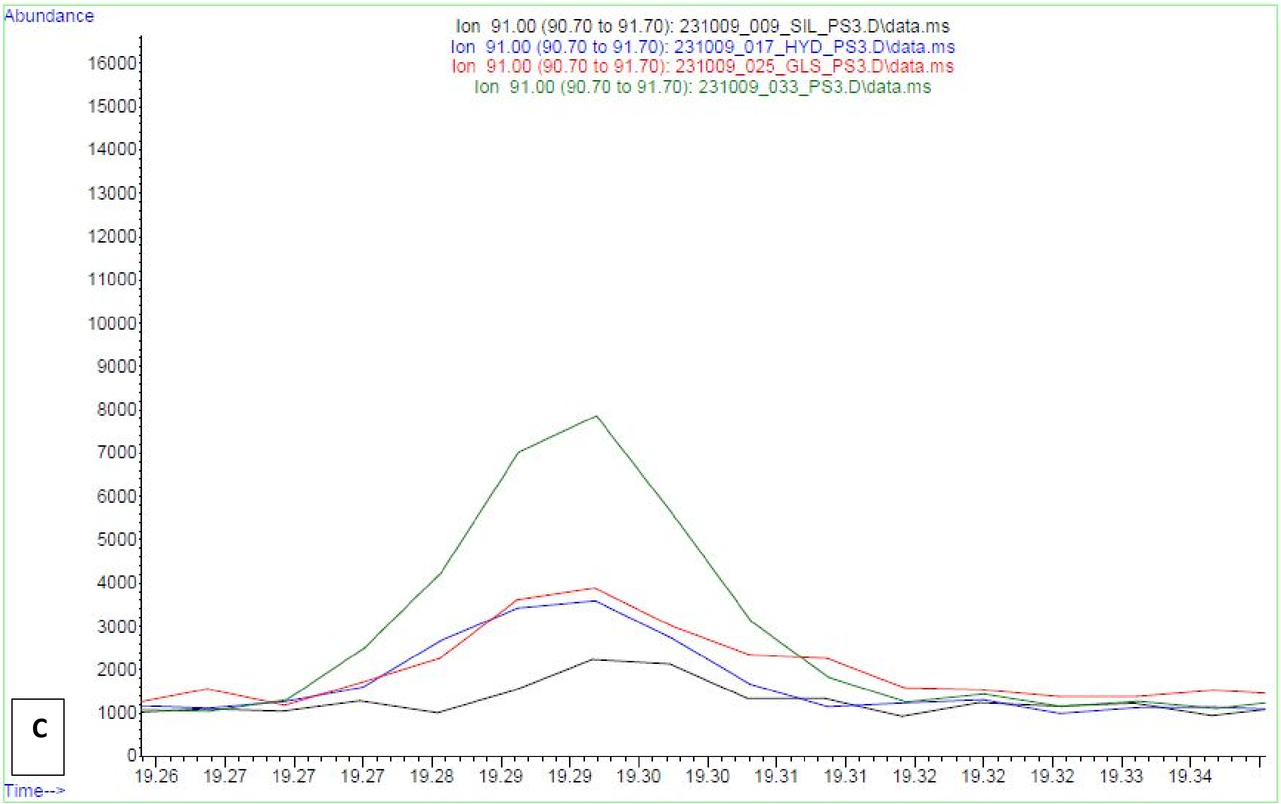

Figure S6: Overlayed chromatogram peak of monomer (m/z 78) (A) , 3-butene-1,3-diyldibenzene (m/z 208) (B) and 5-hexene-1,3,5-triyltribenzene (m/z 91) (C) for PS pyrolysis with different inorganic matrices: silica gel, Hydromatrix, glass beads, and no matrix

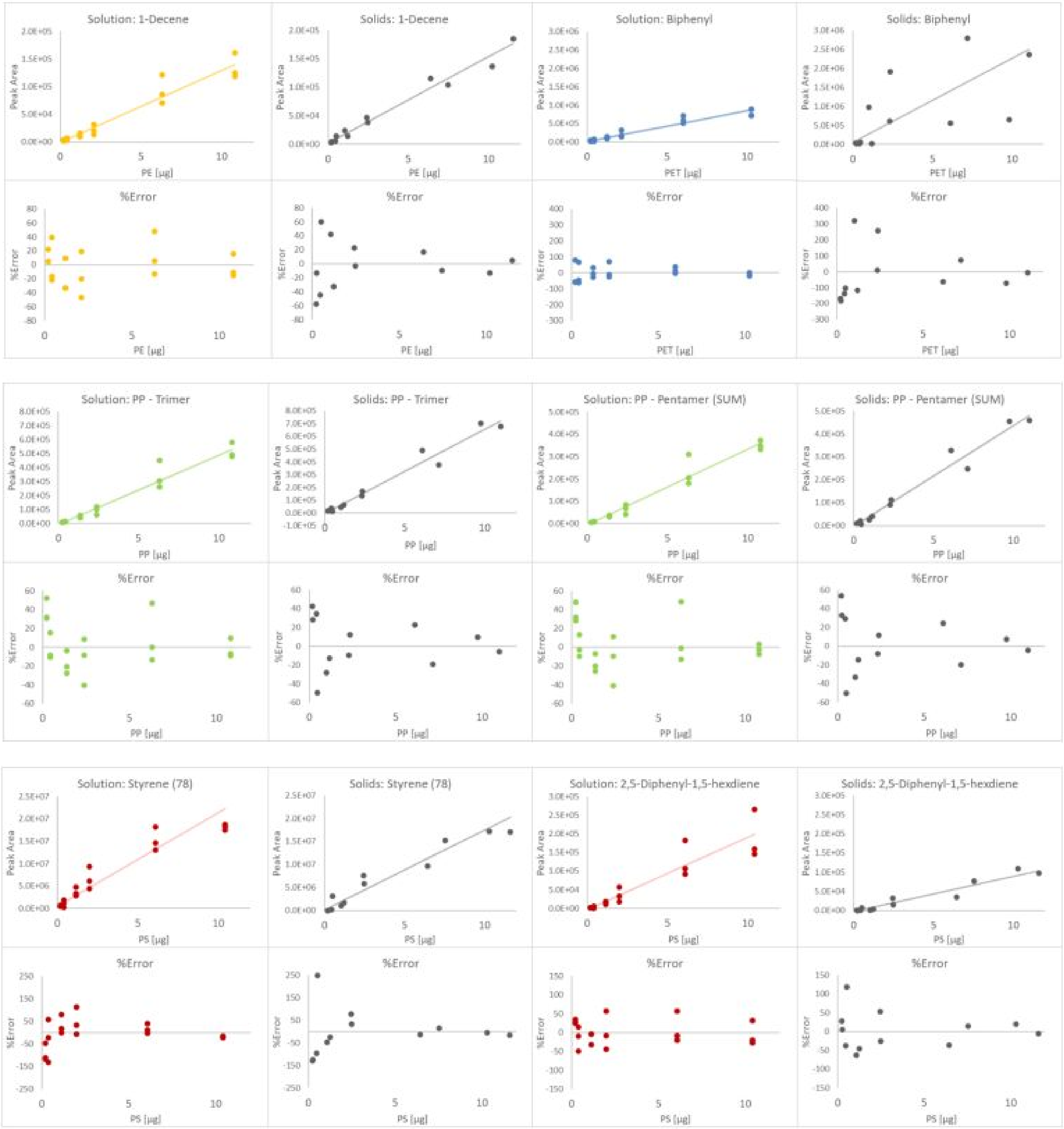

Figure S7: Linear calibration curves, data points, and % error plots for 1-decene, biphenyl, PP trimer, PP pentamer (sum of three peaks), styrene, and 2,5-diphenyl-1,5-hexadiene, comparing solubilized and solid MP calibrations.

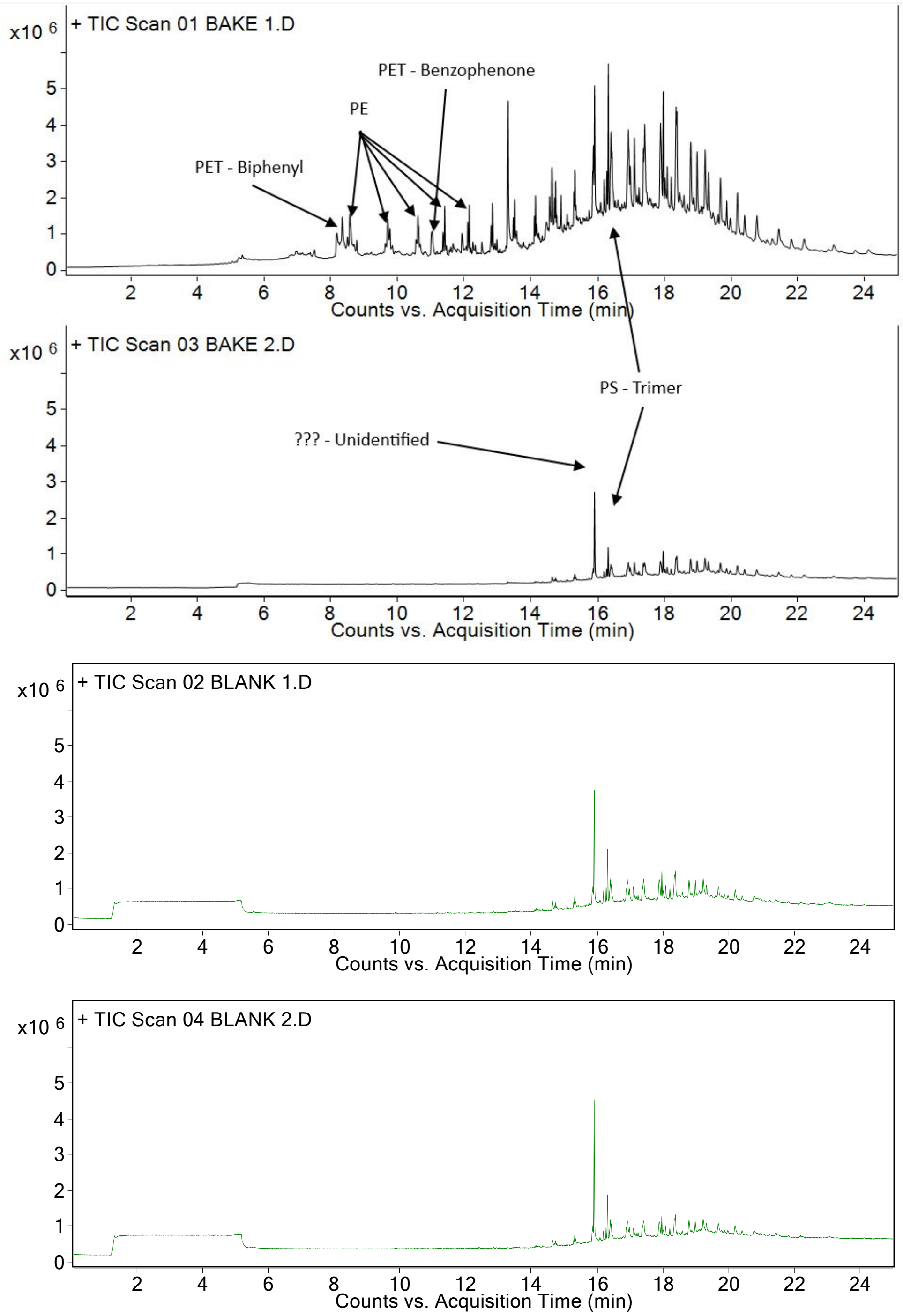

Figure S8: TICs from TDU cleaning process before sample analysis, with TDU heated to 350°C for 30 min followed by blank analysis, performed twice ("TIC Scan 01 BAKE 1" and "TIC Scan 02 BLANK") in MS scan mode, resulting in four pyrograms locked to the same y-axis scale to assess residual signals and cleaning efficiency.

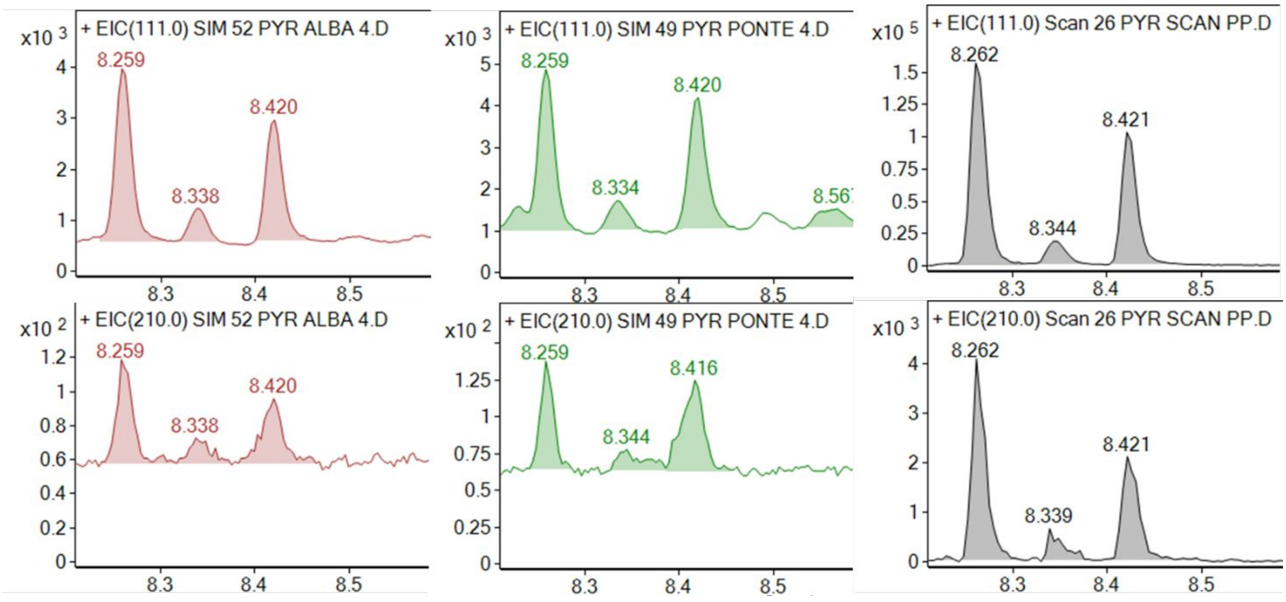

Figure S9: EICs of m/z=111 (quantifier ion for PP pentamer, top row) and m/z=210 (qualifier ion, bottom row) for Alba, Ponte, and pure PP samples, showing peak alignment and presence of three expected peaks for the PP pentamer.

References

(1) Cutroneo, L.; Reboa, A.; Besio, G.; Borgogno, F.; Canesi, L.; Canuto, S.; Dara, M.; Enrile, F.; Forioso, I.; Greco, G.; Lenoble, V.; Malatesta, A.; Mounier, S.; Petrillo, M.; Rovetta, R.; Stocchino, A.; Tesan, J.; Vagge, G.; Capello, M. Microplastics in Seawater : Sampling Strategies , Laboratory Methodologies , and Identification Techniques Applied to Port Environment. **2020**, 8938–8952.

(2) Lindeque, P. K.; Cole, M.; Coppock, R. L.; Lewis, C. N.; Miller, R. Z.; Watts, A. J. R.; Wilson-McNeal, A.; Wright, S. L.; Galloway, T. S. Are We Underestimating Microplastic Abundance in the Marine Environment? A Comparison of Microplastic Capture with Nets of Different Mesh-Size. *Environ. Pollut.* **2020**, *265*, 114721.

(3) Nihan, Ü. Microplastics in a Dam Lake in Turkey : Type , Mesh Size Effect , and Bacterial Biofilm Communities. **2020**, 45688–45698.

(4) Matsueda, M.; Mattonai, M.; Iwai, I.; Watanabe, A.; Teramae, N.; Robberson, W.; Ohtani, H.; Kim, Y. M.; Watanabe, C. Preparation and Test of a Reference Mixture of Eleven Polymers with Deactivated Inorganic Diluent for Microplastics Analysis by Pyrolysis-GC–MS. *J. Anal. Appl. Pyrolysis* **2021**, *154* (August 2020), 104993.

(5) Steinmetz, Z.; Kintzi, A.; Muñoz, K.; Schaumann, G. E. Journal of Analytical and Applied Pyrolysis A Simple Method for the Selective Quantification of Polyethylene , Polypropylene , and Polystyrene Plastic Debris in Soil by Pyrolysis-Gas Chromatography / Mass Spectrometry. *J. Anal. Appl. Pyrolysis* **2020**, *147* (December 2019), 104803.

(6) Okoffo, E. D.; Ribeiro, F.; O'Brien, J. W.; O'Brien, S.; Tscharke, B. J.; Gallen, M.; Samanipour, S.; Mueller, J. F.; Thomas, K. V. Identification and Quantification of Selected Plastics in Biosolids by Pressurized Liquid Extraction Combined with Double-Shot Pyrolysis Gas Chromatography–Mass Spectrometry. *Sci. Total Environ.* **2020**, *715* (January), 136924.

(7) Howell, J.; Roesing, M.; Boucher, D. A Functional Approach to Solubility Parameter Computations. **2017**.
